# Mechanism of proton-powered c-ring rotation in a mitochondrial ATP synthase

**DOI:** 10.1073/pnas.2314199121

**Published:** 2024-03-07

**Authors:** Florian E. C. Blanc, Gerhard Hummer

**Affiliations:** ^a^Department of Theoretical Biophysics, Max Planck Institute of Biophysics, Frankfurt am Main 60438, Germany; ^b^Institute for Biophysics, Goethe University Frankfurt, Frankfurt am Main 60438, Germany

**Keywords:** ATP synthase, c-ring, rotary motor, molecular dynamics simulations, bioenergetics

## Abstract

Proton-powered c-ring rotation in mitochondrial ATP synthase is crucial to convert the transmembrane protonmotive force into torque to drive the synthesis of adenosine triphosphate (ATP). Capitalizing on recent cryo-EM structures, we aim at a structural and energetic understanding of how functional directional rotation is achieved. We performed multi-microsecond atomistic simulations to determine the free energy profiles along the c-ring rotation angle before and after the arrival of a new proton. Our results reveal that rotation proceeds by dynamic sliding of the ring over the a-subunit surface, during which interactions with conserved polar residues stabilize distinct intermediates. Ordered water chains line up for a Grotthuss-type proton transfer in one of these intermediates. After proton transfer, a high barrier prevents backward rotation and an overall drop in free energy favors forward rotation, ensuring the directionality of c-ring rotation required for the thermodynamically disfavored ATP synthesis. The essential arginine of the a-subunit stabilizes the rotated configuration through a salt bridge with the c-ring. Overall, we describe a complete mechanism for the rotation step of the ATP synthase rotor, thereby illuminating a process critical to all life at atomic resolution.

## Significance

Adenosine triphosphate (ATP), the energy currency of living cells, is synthesized by ATP synthase. We show how the membrane potential created by oxidizing food stuff in mitochondria drives the directional rotary motion of this enzyme complex. From molecular dynamics simulations, we determine the free energy surfaces for the rotation of its membrane-anchored c-ring in different protonation states. We capture rotation intermediates as local minima, including the state in which the ring is loaded with a new proton. Strong electrostatic interactions between conserved residues ensure directional c-ring rotation, which is crucial for ATP synthase to function as a molecular machine. By clarifying key steps in ATP synthase rotation, we illuminate a process critical to all life.

**Fig. 1.**
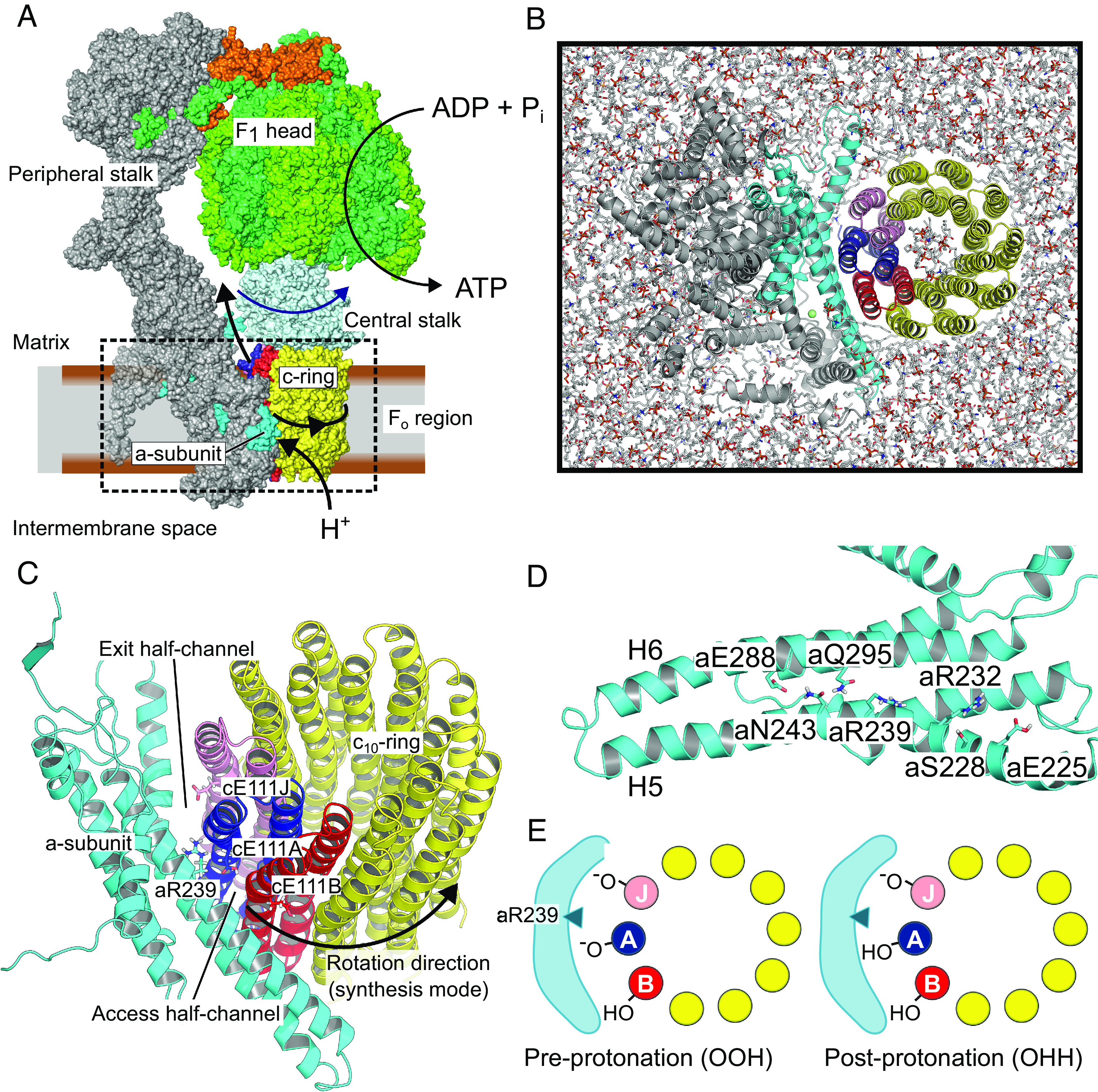
Overview of the system. (*A*) Structure and function of mitochondrial ATP synthase. The box encloses the $F_{o}$ region investigated here. (*B*) Simulation model of *Polytomella sp.* mitochondrial ATP synthase $F_{o}$ region, embedded in a realistic inner mitochondrial membrane (seen from $F_{1}$). For clarity, solvent molecules and ions are not shown. (*C*) Side view of the a-subunit/c-ring complex highlighting important side chains at the interface. (*D*) Inner surface of the a-subunit highlighting the conserved polar residues. (*E*) Schematic of the two protonation states of the c-ring glutamates considered in this study. The drawings also illustrate the naming convention of c-ring subunits. Conventionally, the a-subunit is displayed in cyan; c-ring subunit J in pink; c-ring subunit A in blue; c-ring subunit B in red; all other c-ring subunits in yellow. This color scheme is used throughout the paper.

Adenosine triphosphate (ATP) is the energetic currency powering virtually all cellular processes. Eukaryotic cells produce most of their ATP by mitochondrial respiration. The oxidation of food stuff drives the pumping of protons across the inner mitochondrial membrane (IMM) into the intermembrane space, which results in the build-up of an electrochemical potential, the so-called proton-motive force. The ATP synthase enzyme complex (Fig. 1*A*) sits in the IMM and harnesses the proton-motive force to catalyze the synthesis of ATP (1). The present work is concerned with understanding how proton transfer across the IMM in turn drives the rotation of the transmembrane domain of ATP synthase.

ATP synthase combines two molecular motors. The transmembrane $F_{o}$ region channels the proton flow across the otherwise impermeable IMM in such a way that spontaneous proton transfer powers unidirectional rotation of the turbine-shaped c-ring subdomain. The resulting torque is transmitted to the central stalk domain, whose rotation within the globular $F_{1}$-ATPase domain triggers the conformational changes that power ATP synthesis. The peripheral stalk connects $F_{o}$ to $F_{1}$ and holds $F_{1}$ in place. Given its central importance for cellular bioenergetics and the exquisite complexity of its two coupled rotary motors, ATP synthase has attracted considerable attention throughout decades of biophysical research (1–3). Notably, the rotational mechanism of the central stalk and the $F_{1}$ ATPase motor has been extensively studied (4), including by quantitative molecular simulations initiated from high-resolution crystal structures (5–10).

More recently, the emergence of detailed structural information (11–21) has firmed up a general $F_{o}$ rotational mechanism. The $F_{o}$ rotor, or c-ring, rotates past the membrane-embedded a-subunit (Fig. 1*C*). Their interface harbors two solvated half-channels for protons: the access channel on the intermembrane side and the exit channel on the matrix side. Crystal structures of isolated c-rings in a lipid environment along with biochemical studies have shown that a conserved acidic residue (residue E111 of the c-ring subunit, or cE111 in short, in *Polytomella sp.*) is positioned to act as a proton shuttle. A proton binds to a negatively charged cE111 exposed in the access channel. Upon protonation, the now neutral glutamate can insert into the membrane, promoting rotation of the whole c-ring by one elementary step. Successive elementary rotations eventually lead the protonated glutamate to be exposed to the mitochondrial matrix in the exit channel. There, membrane voltage and higher pH conditions promote proton abstraction. Finally, one last elementary rotation puts the glutamate side chain back in the access channel.

Still lacking are a detailed and comprehensive picture of the substeps in the overall rotation mechanism and, even more importantly, a quantitative representation of the energy landscape underlying proton-driven c-ring rotation. Molecular Dynamics (MD) simulations of the c-ring at the atomistic and coarse-grained levels have advanced our understanding of the proton transfer process through the membrane and of the coupled rotation of the c-ring (22–25). However, structural information on the critical a-subunit has emerged only recently. Cryo-EM structures of full-length ATP synthase complexes at high resolution, including the a-subunit, now open the way to a detailed analysis of the rotation mechanism by molecular simulations (26–32).

Here, we probe the structural rearrangements and free energy profiles along the rotation of the c-ring of *Polytomella sp.* mitochondrial ATP synthase in an all-atom $F_{o}$ model embedded in a realistic IMM (Fig. 1*B*) (11, 16, 33). To understand how c-ring rotation is effected by the shifting protonation landscape of the a/c interface (Fig. 1*D*), we compute the free energy profiles of an elementary c-ring rotation for the two possible protonation states of c-ring proton-carrying glutamates, namely before and after protonation (Fig. 1*E* and *SI Appendix*, Tables S1 and S5). Our results provide a plausible mechanistic and energetic description of the “electro-osmo-mechanical” step in ATP synthase operation.

## Results

Only two c-ring protonation states are likely to be relevant for proton-driven c-ring rotation (Fig. 1*E*). The OOH state has the two adjacent c-ring subunits straddling the critical arginine unprotonated. By contrast, the OHH state has only one c-ring subunit unprotonated. By the 10-fold symmetry of the *Polytomella* $c_{10}$-ring, the free energy surface of the OHH state is equivalent to the free energy surface of an HOH state shifted by 36^°^. The two relevant rotational free energy surfaces to be calculated, for the OOH and OHH states, are connected by proton transfer reactions, which will not be studied explicitly here. We assume that the rotary free energy surfaces are largely unaffected by any transmembrane potential difference because charges move parallel to the membrane plane in all processes modeled here.

### Free Energy Profiles along the c-ring Rotation Angle.

**Fig. 2.**
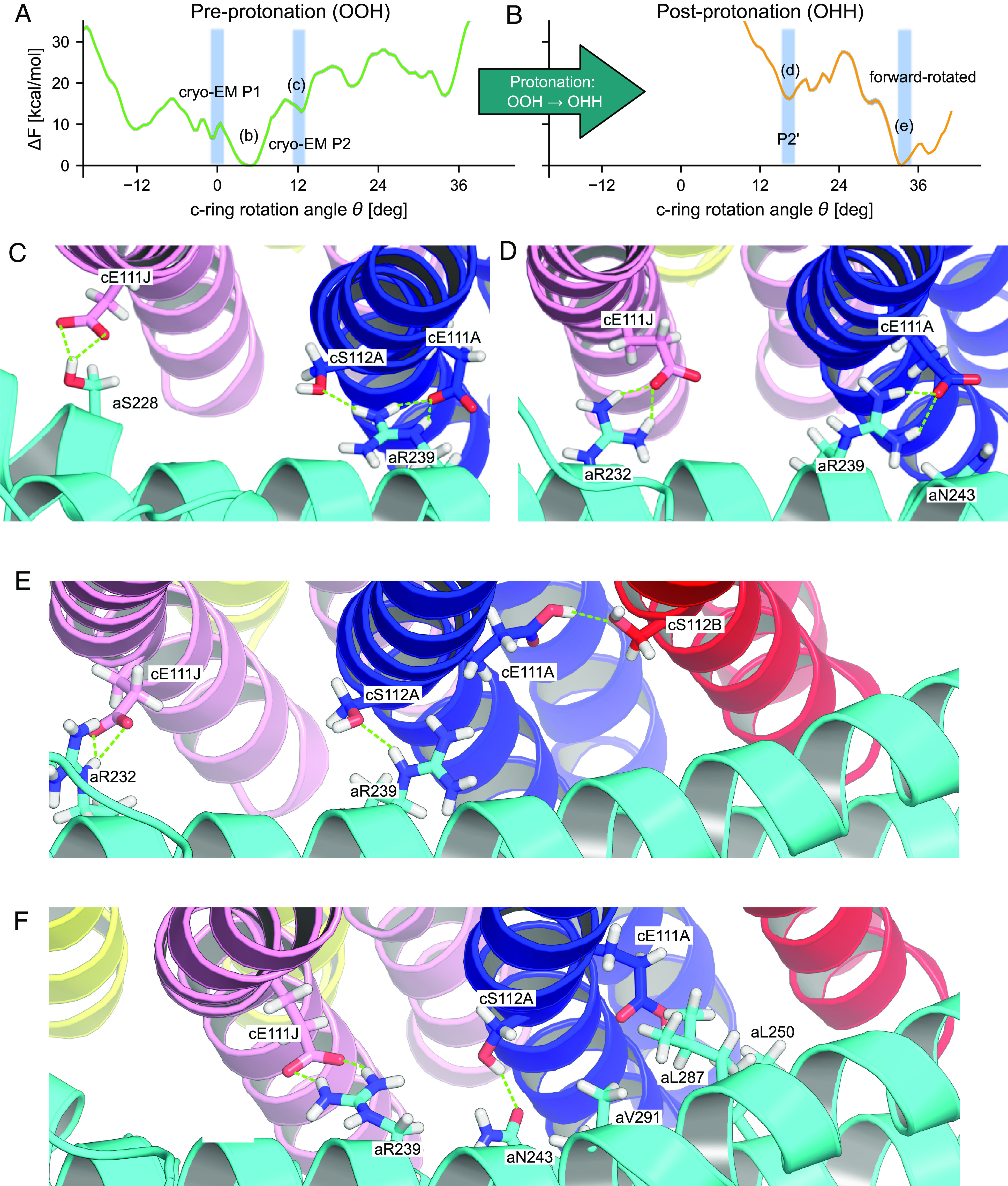
Free energy profiles and relevant configurations along c-ring rotation. (*A* and *B*) PMF along θ for the pre-protonation state OOH (*A*) and post-protonation state OHH (*B*). Gray outlines indicate ± statistical error estimated by bootstrap (*SI Appendix*, Fig. S3). Shadings indicate relevant metastable states including cryo-EM structures P1 (PDB:6RD7/6RD9) and P2 (PDB:6RD8). (*C*) OOH ground state at $\theta \approx 5^{{}^{\circ}}$. (*D*) OOH substate P2 at $\theta \approx 12^{{}^{\circ}}$. (*E*) OHH substate P2’ at $\theta \approx 16^{{}^{\circ}}$. (*F*) OHH forward rotated state at $\theta \approx 36^{{}^{\circ}}$.

We used geometric free energy calculations with the extended Adaptive Biasing Force (eABF) method (34–38) and a stratification strategy to estimate the potentials of mean force (PMFs) along the rotation angle θ of the c-ring (Fig. 2), *i.e.,* the angle-dependent free energy profiles. Moreover, we augmented the natural reaction coordinate θ with a second coordinate, namely the distance $d_{1}$ between the cE111J CD atom and the aR239 CZ atom. $d_{1}$ accounts for the possible formation of a salt bridge between these two residues as rotation proceeds, allowing us to probe the thermodynamics of this functionally relevant interaction. Additionally, biasing this orthogonal degree of freedom should favor convergence of the calculations.

Our calculations, performed separately for the two relevant protonation states, OOH and OHH (Fig. 1*B*–*E*), represent nearly ∼70μs of all-atom MD simulation (*SI Appendix*, Tables S2). The description and comparison of the profiles before and after protonation of the c-ring glutamate provide insight into the sequence of structural rearrangements, the plausible timing of proton transfer, and the energetics of the process.

For both relevant protonation states, the free energy profiles along θ exhibit several metastable states, including some that are separated by ${\approx 36}^{{}^{\circ}}$, *i.e.,* the size of an elementary rotary step (Fig. 2*A*). Therefore, the PMFs are consistent with the expected spacing of the rotation free energy landscape, showing that our eABF calculations did pick up this important feature of the system. This point, along with the full, nearly uniform coverage of configurational space (*SI Appendix*, Fig. S1 *C* and *D*), the convergence of the force estimate (*SI Appendix*, Fig. S2) and the low statistical error estimates (*SI Appendix*, Fig. S3), supports the proper convergence of these challenging sampling problems.

#### Pre-protonation state OOH.

State OOH exhibits a ground state at $\theta =5^{{}^{\circ}}$, *i.e.,* with a slight forward rotation with respect to the reference configuration (Fig. 2*A*). This finding indicates that already the deprotonation of state HOH contributes to the forward rotation of the c-ring. The reference cryo-EM structure P1, *i.e.,* $\theta \approx 0^{{}^{\circ}}$, corresponds to a metastable state and belongs to the same overall basin as the $5^{{}^{\circ}}$ state. From this basin, rotation in both directions entails an increase in free energy, corresponding to the cost of breaking the strong cE111A:aR239 interaction and inserting charged glutamate side chains into the hydrophobic membrane. This is another expected feature of the free energy landscape which we correctly capture. Finally, a local minimum identified at $\theta \approx 12^{{}^{\circ}}$ matches the so-called P2 alternate ring position observed in cryo-EM (33). Overall, the free energy profile for our $F_{o}$ system thus captures two key structures as local minima, indicating at least semi-quantitative consistency with experiments on the full $F_{o}$$F_{1}$ system.

To provide a structural interpretation of the observed metastable states (Fig. 2 *C* and *D*), we computed the contact probability maps between key residues of the a and c subunits over the concatenated eABF trajectories. The contact maps of residues cE111 of c-ring chain A (thereafter, cE111A, which is followed by cE111B to cE111J around the ring) and cE111J with respect to polar a-subunit residues show that metastable states match reasonably well with specific, stabilizing contacts being formed (*SI Appendix*, Fig. S4 *A*, *C*, and *E*). For example, the pre-protonation ground state at $5^{{}^{\circ}}$ is associated with a cE111J-aS228 hydrogen bond and a salt bridge between cE111A-aR239, which are likely to be the main contributors to the stability of this substate (Fig. 2*C*). Similarly, in typical configurations, substate P2 is stabilized by a cE111J:aR232 salt bridge and interactions of cE111A with aN243 and aR239 (Fig. 2*D*), and is also compatible with configurations where cE111J replaces cE111A as the main interaction partner of aR239 (*SI Appendix*, Fig. S1*A*). Therefore, the existence of a sequence of intermediates along the rotation pathway follows from the structural organization of the a/c interface, in which polar residues are positioned to sequentially form transient interactions.

#### Post-protonation state OHH.

The overall minimum of the OHH rotational PMF is at $\theta \approx 32^{{}^{\circ}}$ to $36^{{}^{\circ}}$ (Fig. 2*B*). Considering the 10-fold symmetry, we can shift the OHH surface to the left by $36^{{}^{\circ}}$ to obtain the equivalent HOH surface, which thus has a minimum close to the most populated experimental angular state of $0^{{}^{\circ}}$. In addition, the post-protonation free energy profile exhibits several local minima. In comparison to the pre-protonation profile OOH, it shows a strong downhill trend to the forward-rotated state at $\theta \approx 36^{{}^{\circ}}$ (Fig. 2*B*). This downhill nature of the free energy profile results from the increasing electrostatic attraction between cE111J and aR239 (described by $d_{1}$, see *SI Appendix*, Fig. S1) as rotation proceeds, and is also consistent with the more favorable insertion of the now neutralized cE111A into the membrane. Thus, the difference between the OOH and OHH PMFs clearly shows how protonation promotes rotation by shifting the free energy landscape, supporting our assumption that state OHH is primed for rotation.

The finer structure of the free energy profile, namely intermediates and barriers, nonetheless shows that the completion of the elementary rotation step does not proceed solely through relaxation along the free energy gradient, but involves a sequence of thermally activated transitions. Similar to the pre-protonation state, we used a contact map analysis to interpret these on-pathway intermediates in terms of a/c polar interactions (Fig. 2 *E* and *F*). We found that the final forward rotated state is stabilized by the cE:aR salt bridge, established between the charged cGlu on the trailing c-ring subunit (cE111J) and the essential, absolutely conserved aArg239 residue of the a-subunit (Fig. 2*F*). Additional stabilization to the forward rotated state is provided by a hydrogen bond between aN243 and cS112A. Interestingly, in this state, the protonated cE111A side-chain still retains hydrating water molecules (*SI Appendix*, Fig. S6*C*) and can either face toward the a-subunit inner surface or be in the so-called “ion-locked” configuration (39). In this configuration, cE111A flips away from the a/c interface and forms a hydrogen bond with cS112B (*SI Appendix*, Fig. S4*D*). A hydrophobic cluster on the a-subunit (aL250, aL287, aV291, see Fig. 2*F*) may also contribute to the high free energy cost of rotating a charged c-ring subunit past $\theta \approx 12^{{}^{\circ}}$ to $16^{{}^{\circ}}$.

Finally, we note that protonation destabilizes state P2, which is no longer a free energy minimum; instead, a novel minimum appears at $\theta =16^{{}^{\circ}}$, which we name P2’ (Fig. 2*E*). Protonation of cE111A destabilizes the cE111A:aR239 salt bridge and makes cS112A the main interaction partner of aR239. As a result, the c-ring rotates forward by ${+4}^{{}^{\circ}}$ and cE111A takes on the ion-locked configuration. Further comparison of P2 and P2’ reveals that no other major conformational rearrangement occurs upon protonation of the rotor (*SI Appendix*, Fig. S5).

### Hydration of the a/c Interface.

In agreement with high-resolution cryo-EM structures (20, 33) and previous MD simulations (28, 30, 31, 40), we observe that water populates the access and exit half-channels all the way to the a/c interface (*SI Appendix*, Fig. S6*A*). Water permeates the access half-channel through an opening between a-subunit H5 and H6 leading up to aH248 and aE288. Interestingly, the leading cGlu side chain (cE111A) remains solvated in the ${+36}^{{}^{\circ}}$ forward-rotated state for both OOH and OHH (*SI Appendix*, Fig. S6 *C* and *D*). Furthermore, cGlu side chains buried deeply in the membrane are still marginally hydrated (subunits C-H, *SI Appendix*, Fig. S6 *C* and *D*). These observations suggest that desolvation of the protonated cGlu is not a strict requirement for membrane insertion as rotation proceeds. This may result in marginal transmembrane water flux, but not proton leakage because of the charge penalty. Lipid molecules, including cardiolipin, border the a-subunit on each side and may contribute to the interface (*SI Appendix*, Fig. S8).

### Proton Transfer and Role of State P2.

Whereas our classical simulations cannot directly describe the covalent chemistry of protonation of the c-ring acceptor residue cE111A, the analysis of interfacial water molecules in the eABF trajectories provides us with a plausible model for the timing of the proton transfer steps. The main result emerging from this analysis is that P2 is the most plausible rotational state for protonation of the c-ring to take place through a Grotthuss mechanism.

#### Direct proton transfer is unlikely.

**Fig. 3.**
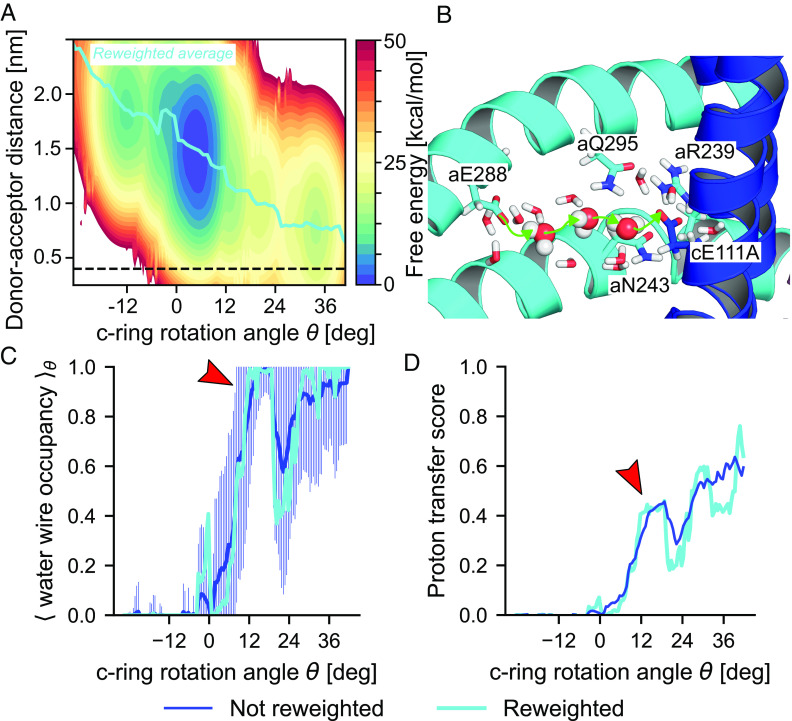
Facile protonation of the c-ring requires rotation into P2 state and water bridge. (*A*) PMF as function of rotation angle θ and distance between proton donor aE288 and acceptor cE111. The thick cyan line indicates the mean distance as function of θ, and the horizontal dashed line the distance where a direct proton transfer would be feasible. (*B*) Representative bridge of three water molecules between aE288 to cE111A in state P2. (*C*) Occupancy of water wires of length ≤8 in state OOH. Shown are the eABF-reweighted average (cyan) and nonreweighted average (blue) of the indicator function, which is equal to 1 if a wire of length ≤8 exists in the trajectory frame, 0 otherwise (shading: SD). (*D*) Water-mediated proton transfer score computed from eABF simulations of the pre-protonation state as function of θ (cyan: eABF-reweighted; blue: nonreweighted). Red arrows in (*C* and *D*) indicate state P2.

For *E. coli* ATP synthase, mutagenesis studies support aH245 as the proton donor (41). In view of the conservation of the a-subunit (3), we assume an equivalent role for its *Polytomella sp.* homolog aE288 (33). To assess the possibility of proton transfer by direct side-chain/side-chain interaction, we computed the 2-dimensional free energy profile $F(\theta ,d_{\;\text{donor-acceptor}})$ (Fig. 3*A*) by reweighting the eABF trajectories of state OOH (*SI Appendix*, *Text*). We also evaluated, as a function of θ, the (reweighted) average donor-acceptor distance. The results clearly demonstrate that direct contact between side chains aE288 and cE111 (<4Å) is essentially never seen in the OOH state (Fig. 3*A*). Because our eABF calculations were performed with a deprotonated aE288 (*Discussion* and *SI Appendix*, *Text*), we then sought to evaluate whether protonating aE288 could favor direct aE288-cE111 contact. For this purpose, we ran θ-restrained MD simulations of frames extracted from the OOH eABF simulation, to which we added a proton on aE288 (*SI Appendix*, *Text*). The θ-conditional averages and minimal values of the donor-acceptor distance measured from these simulations confirm that aE288 and cE111 do not come close enough for direct proton transfer in accessible states (*SI Appendix*, Fig. S7*A*). We conclude that the c-ring is not protonated directly as it rotates past aE288. As an alternative, the proton may be transferred via water wires in a Grotthuss-type mechanism (42), as recently proposed by Spikes et al. (20).

#### Grotthuss-type proton transfer along water wires is possible in state P2.

Hydration of the acceptor residue (cE111A) is a necessary condition for water-mediated proton transfer, but is not sufficient. Water wires connecting the proton donor (aE288) and acceptor residues could provide a pathway for proton transfer. Therefore, we searched for water wires in the OOH trajectories that connect these two side chains by analyzing hydrogen bond networks between water molecules in their vicinity. This analysis clearly shows that water wires of length ≤8 are virtually always formed around state P2 ($\approx 11^{{}^{\circ}}$ to $16^{{}^{\circ}}$), whereas they reach only ≈20% occupancy in the ground state (Fig. 3*C*). Further, wires of length 4 or lower are frequently observed for $\theta \geq 12^{{}^{\circ}}$, whereas they are very rarely seen at lower values of the rotation angle (*SI Appendix*, Fig. S5*B*). An example of a length-3 wire in state P2 is shown in Fig. 3*B*. Although water wires are occasionally observed for negative θ states, they are very long (≥7 water molecules on average) and extend past the positively charged aR239 which separates the two half-channels, blocking proton leaks. Therefore, these wires will not be conducive to proton transfer. Conversely, for $\theta \geq 0^{{}^{\circ}}$, the leading c-ring subunit (A) is located in the access half-channel and proton transfer would thus not be impeded by aR239. In these conditions, proton transfer along a single-file water wire is contingent upon every involved water molecule adopting a favorable orientation, which becomes rate-limiting, with the actual proton transfer occurring within femtoseconds (43). Thus, the efficiency of proton transfer along a water wire is expected to decrease geometrically with wire length, provided a wire exists in the first place (44). These considerations can be formalized into a proton-transfer score (*SI Appendix*), which we used as a summarizing descriptor for the efficiency of c-ring protonation as a function of θ. The θ-dependent proton-transfer score evaluated from the eABF simulations of state OOH shows that P2 represents the first intermediate state to exhibit water wires both short enough to be conducive to proton transfer, and occurring with significant occupancy probability. Evaluation of these observables on the θ-restrained simulations with protonated aE288 leads to the same conclusions, although the occupancy of water wires decreases slightly (*SI Appendix*, Fig. S7 *B*–*E*). From these simulations, we can also evaluate the typical lifetime of water wires in state P2 to ≈100ns, further suggesting they can support proton transfer (*SI Appendix*, *Text* and Fig. S7*F*. We therefore conclude that water-mediated proton transfer is maximally probable in state P2, which ideally represents the proton-transferring state. Thus, we have an experimental structure for further examination of this important chemical step (33). We also note that small but nonzero proton-transfer scores are observed for $\theta \geq 5^{{}^{\circ}}$, suggesting a probabilistic picture in which proton transfer takes place over a range of angular states, with P2 nonetheless maximizing the protonation probability.

### Dynamics of the *Zn*^2+^ Cation.

An electron density in the cryo-EM map of *Polytomella sp.* near residue aH248 was attributed to a $Zn^{2+}$ ion, which takes slightly different positions in cryo-EM states P1 and P2 (33), see *SI Appendix*, Fig. S7*A*. We performed blind prediction using a state-of-the-art convolutional neural network on the P1 structure, which recovered the assigned Zn binding site, supporting the attribution decision (45). Because this cation may be involved in proton transfer (33), we investigated its positional dynamics and hydration shell in eABF simulations of state OOH (*SI Appendix*, Fig. S9 *A*–*G*). We found that the $Zn^{2+}$ spatial distribution depends on θ. In the ground state, fluctuations of amplitude ≈5 Å in the z direction are observed (*SI Appendix*, Fig. S9*B*), along with higher variance in the number of water molecules in the hydration shell (*SI Appendix*, Fig. S9*C*). In P2, the fluctuations narrow down, and the cation interacts more closely with aE172 and aH248. Therefore, our findings suggest that the rotational state of the c-ring influences the positional dynamics of ${\mathrm{Zn}}^{2+}$, in qualitative agreement with the cryo-EM observation that the cation moves from P1 to P2.

## Discussion

We propose a complete mechanism of ATP synthase c-ring rotation, using all-atom free energy simulations representing nearly 70μs of accrued simulation time. Our results shed light on important open questions about the proton-powered rotation of the $F_{o}$ motor of *Polytomella sp.* mitochondrial ATP synthase. Because the general architecture of ATP synthase is conserved, we discuss our findings in light of recent structural, single-molecule, and computational studies of various ATP synthase isoforms including *E. coli*, yeast mitochondria and *Bacillus sp.* PS3.

**Fig. 4.**
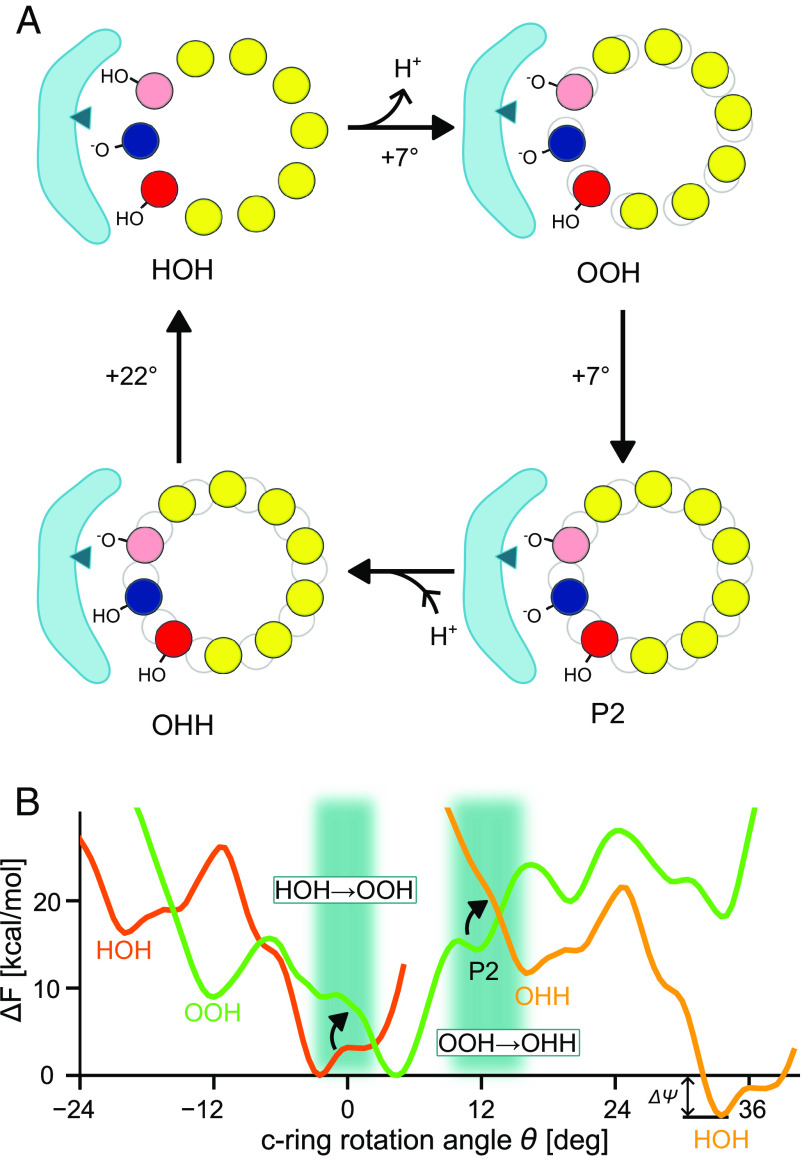
Mechanism of proton-driven $F_{o}$ rotation. (*A*) Cycle of coupled proton transfer reactions and c-ring rotations. For easier visualization of rotation movements, the gray outline materializes the initial position of the c-ring. (*B*) Free energy landscape of c-ring rotations coupled to proton transfer onto and off the rotating c-ring. Free energy profiles for states HOH and OHH are identical, up to a vertical shift by $\Delta \psi =4.6\;\mathrm{kcal}\;{\mathrm{mol}}^{-1}$ (*i.e.,* the value of the protonmotive force) and horizontal shift by $36^{{}^{\circ}}$. Shaded rectangles indicate proton transfer events.

The rotation mechanism emerging from our analyses is summarized in Fig. 4*A*. Initially, the motor is in the HOH state. In the exit half-channel, cE111J releases its proton, to bulk water or possibly a proton-accepting residue on the a-subunit such as aE225. The resulting OOH state rotates forward by $\theta \approx 5^{{}^{\circ}}$ to $7^{{}^{\circ}}$ due to the rearrangement of polar contacts. The c-ring then undergoes angular fluctuations, until a thermally activated transition to state P2 is captured. This uphill transition over a significant free-energy barrier probably represents the rate-limiting step. We note that the barrier of $16\;\mathrm{kcal}\;{\mathrm{mol}}^{-1}$ is likely overestimated because of imperfect sampling of the conformational dynamics. Then, protonation of the c-ring takes place in P2 along a structured water wire. This shifts the free energy landscape and destabilizes P2, promoting the rapid relaxation of the c-ring toward a metastable state at $\theta =16^{{}^{\circ}}$. Finally, angular fluctuations allow the c-ring to cross smaller free energy barriers until the forward-rotated state is reached. Because of the large free energy gradient, this substep contributes most of the torque developed during c-ring rotation. Its driving force is the formation of the cE111J:aR239 salt bridge, which provides a strong stabilization to the forward-rotated state, *i.e.,* HOH, and completes the elementary rotation step. Thus, this mechanism has features both of a powerstroke (down-gradient relaxation) and a Brownian ratchet (“capture” of forward rotational fluctuations) (46).

Our calculations also yield a rough prediction of the maximum torque Γ developed by proton-powered c-ring rotation. The torque-producing free energy drop evaluates to $\Delta F\approx -18\;\mathrm{kcal}\;{\mathrm{mol}}^{-1}$. Taking Γ∼ΔF/Δθ with $\Delta \theta =36^{{}^{\circ}}$ the total angular amplitude, we find Γ≈200pNnm. This is only a factor 5 higher than the 40pNnm torque measured experimentally in *E. coli* (47).

Experimental mutagenesis identified aE288 as the final proton donor, suggesting that c-ring protonation may take place in a rotational state where cE111A and aE288 side chains are close enough for direct proton transfer (41). However, our calculations suggest that such states may not be accessible from the pre-protonation state and instead support the growing consensus that incoming protons are transferred to the c-ring along a water wire through a Grotthuss mechanism (20, 40, 48). Unlike previous computational studies of $F_{o}$, which only evaluated global hydration of cGlu (28, 30), we specifically searched for water wires. Distinct short water wires between donor aE288 and acceptor cE111A indicate P2 as the likely state for proton transfer onto the c-ring. We note in passing that the proton-transfer score we introduce could be used to investigate water-mediated proton transfer in other molecular systems. Upon transition to P2, the $Zn^{2+}$ cation moves toward aE172, away from aH252 and is seemingly held more strongly. In P2, $Zn^{2+}$ may provide an electrostatic barrier to prevent reverse transfer of protons to the bulk when the rotor is geared for protonation. Conversely, in P1, the increased positional freedom of $Zn^{2+}$ may ensure that it does not hinder proton access to aH248 and aE288. The opening state of this “Zn-barrier” would be controlled by the competitive interactions with aE172 and aH252 (*SI Appendix*, Fig. S7 *D*–*G*). This hypothesis is consistent with Murphy et al.’s proposal that Zn helps synchronize proton access to the c-ring in the P2 proton-accepting state (33).

To keep the complexity manageable, the putative proton-donating (aE288, deprotonated) and proton-accepting (aE225, protonated) a-subunit residues were modeled in the same protonation state for both OOH and OHH, which were those predicted by our titration calculations. Accounting for the transient changes in the protonation states of these residues in a full cycle of c-ring rotation coupled to proton transfer may alter the PMFs. However, in *E. coli* ATP synthase the charge environment of both residues could be changed by mutation without significant effect on function (41, 49, 50), suggesting that the effects on the PMFs are small. Additionally, our restrained-c-ring simulations indicate that the proposed water-mediated proton transfer mechanism still holds when aE288 is protonated.

Remarkably, when our protonation-dependent PMFs are shifted vertically by $\Delta \psi =4.6\;\mathrm{kcal}\;{\mathrm{mol}}^{-1}$ (corresponding to a 200mV transmembrane electrochemical potential) and horizontally by ${\pm 36}^{{}^{\circ}}$ (*i.e.,* the angular size of an elementary rotation), they intersect roughly at the presumed proton-transferring states including P2, suggesting that proton transfers are essentially iso-energetic (Fig. 4*B*). Proton transfer would then move the system from the OOH to the OHH surface, where it first relaxes to a local minimum at about $16^{{}^{\circ}}$ before advancing to the new ground state near $36^{{}^{\circ}}$ (Fig. 4*B*).

### Intermediates along the Rotation Pathway.

Our results suggest that a/c interactions stabilize several intermediates along the rotation pathway, including the experimentally characterized P2. Thus, conserved polar residues on the inner surface of the a-subunit (Fig. 1*D*) are positioned to enable successive formation/breaking of interactions between a and c, thereby realizing a dynamic sliding of the c-ring onto the stator by stochastic jumps between metastable states. Whereas a large, negative free energy gradient would thermodynamically promote net directional rotation, it may not be sufficient to reach biologically useful timescales. Instead, by establishing smaller barriers between successive rotation substeps, on-pathway intermediates could facilitate rotation, as proposed for other biological and artificial molecular machines (51). An additional role for the polar residues, which is also supported by our simulations, may be to help stabilize the water wires and thus assist with proton transfer (18, 48), Fig. 3*B*.

### cE:aR Salt Bridge.

Among the conserved residues of the a-subunit, the critical arginine (aR239) is required for proper function of the proton-powered rotor (52–56). The electrostatic interaction cE:aR between cE111 and aR239 was proposed to contribute to directionality (57). A cE:aR salt bridge is observed in some recent cryo-EM structures (20), but not all (3, 17), and its existence has also been challenged in a recent NMR study (58). Our results show that a salt bridge is formed nearly without interruption throughout rotation, either with the leading (A) or trailing (J) c-ring subunit. The transition between the two seems to happen precisely in state P2, which could help orient the cE111A side chain toward the water channel. In the OOH state, the competition between salt bridges involving adjacent cE111 residues transiently stalls the rotor; protonation promotes rotation by favoring the formation of the aR239:cE111J interaction.

### Nature of the State Primed for Rotation.

Whether a single-charged (OHH) or double-charged (OOH) ring-protonation state is primed for rotation is unclear. Triple-charged (OOO) intermediates were observed in coarse-grained simulations of c-ring rotation with dynamical protonation, lending credit to an OOO/OOH scenario (27). Here, the comparison of the energetics of forward rotation reveals that only state OHH exhibits a large free energy drop for forward rotation, consistent with it being the primed state. By contrast, in state OOH, forward rotation clearly entails an increase in free energy, making forward-directed rotation from this state unlikely. Given the three charged cGlu, the PMF for state OOO would also exhibit steep climbs in free energy in both rotation directions. Therefore, in the OOO/OOH scenario, there is no state with the expected characteristics of the primed state. Our model relies on the hypothesis of OHH and OOH states being most relevant, which is supported by alchemical calculations on yeast ATP synthase indicating that OHH may be the dominant protonation state (28). A recent solid-state NMR study found that up to 4 cGlu side chains are facing the a-subunit, suggesting that 4-times deprotonated (*i.e.,* OOOO) configurations are explored (59). However, our results show that such “open” conformations are also compatible with protonated cGlu (Fig. 2*E*).

### Comparison with Single-Molecule Studies.

Recent single-molecule experiments on *E. coli* ATP synthase concluded that a $36^{{}^{\circ}}$ elementary rotation is broken up into an $11^{{}^{\circ}}$ protonation-dependent step, followed by a $25^{{}^{\circ}}$ rotation driven by electrostatic interaction (48, 60–62). Our results are remarkably consistent with these findings, whose mechanistic underpinnings they firm up. A minor difference is that we find the initial step to be split into two substeps, with deprotonation of the lagging c-ring subunit (HOH → OOH) contributing $5^{{}^{\circ}}$ to $7^{{}^{\circ}}$ and the transition to state P2 $5^{{}^{\circ}}$ to $7^{{}^{\circ}}$. The total extent of this step, $10^{{}^{\circ}}$ to $14^{{}^{\circ}}$, is consistent with the experimental measurement of ${11\pm 3}^{{}^{\circ}}$, all the more so that perfect agreement is not to be expected since the single-molecule experiments were performed with full-length $F_{o}$$F_{1}$ ATP synthase from *E. coli*. Both the sequence differences and the influence of the central stalk and $F_{1}$ head may affect the details of the mechanism. Recent $F_{o}$ high-resolution structures suggest that the $\theta =10^{{}^{\circ}}$ to $14^{{}^{\circ}}$ substep is common in a range of species (18, 33), suggesting that this mechanism could be general.

### Comparison with Earlier All-Atom MD Studies.

Recently, free energy calculations along c-ring rotation were reported for yeast mitochondrial ATP synthase (28), and for *Bacillus PS3* ATP synthase (30, 31). For yeast, state OOH was deduced to undergo rotational diffusion up to ${\approx 10}^{{}^{\circ}}$, which is in line with our findings. The interactions between aR (aR176 in yeast) and other a-subunit polar residues, and the c-ring glutamate (cE59 in yeast) were found to shape the rotational free energy landscape. The leading cGlu got progressively more hydrated as rotation proceeded from θ=0, supporting an increase in protonation probability and an assignment of the ${\approx 10}^{{}^{\circ}}$ configuration as the probable proton-transferring state. Our findings agree with these conclusions. Yet, in contrast to our model, the authors propose that directional rotation stems from the free energy barrier to forward rotation being lower than that for backward rotation in the primed state (OHH) (28). This proposal is based on umbrella sampling and could be consistent with recent theoretical analyses of molecular motors (63). However, the yeast rotational PMF for state OHH shows a steep climb in free energy in both directions and no marked free energy minimum at the forward rotated state. To achieve a decreasing free energy in the synthesis direction, these investigators had to deprotonate c-ring subunit I; however, this site is buried in the membrane and thus unlikely to be accessible for deprotonation. The resulting state is equivalent to our OOH state rotated by ${-36}^{{}^{\circ}}$. Although this state would indeed rotate forward, this movement would not result in a cycle of rotations that can be closed. Thus, this state is unlikely to be representative of a functional rotational state, leaving the rotation mechanism unclear.

1μs-long MD simulations of *Bacillus* PS3 $F_{o}$ led to the conclusion that the Coulombic attraction between the deprotonated trailing cGlu and aR in state OHH is the main driving force for c-ring rotation (30). Further, these investigators deduced that the HOH → OOH → OHH transitions occur with minimal angular change (30). The subsequently reported free energy profile (31) for state OHH exhibits an $\approx 8\;\mathrm{kcal}\;{\mathrm{mol}}^{-1}$ barrier and a negative gradient from ${-20}^{{}^{\circ}}$ to $0^{{}^{\circ}}$, compatible with forward rotation. However, no on-pathway intermediate was detected [but note that the reference c-ring *Bacillus* PS3 structure (19) (PDB: 6N2D) is actually rotated by ${\approx +10}^{{}^{\circ}}$ with respect to the *Polytomella* reference (PDB: 6RD7/6RD9), *SI Appendix*, Fig. S10]. Here, by using the aR239:cE111J salt-bridge distance as an auxiliary reaction coordinate for free energy calculations, we arrive at free energy profiles both for OHH and OOH states that are consistent with forward rotation in a closed cycle (Fig. 4) and capture a key intermediate consistent with structural (20, 33) and single-molecule studies (48, 60, 62, 64).

### Importance of Lipids.

We observe that lipids, including one cardiolipin, populate the outer a/c interface on both the IMS and matrix sides (*SI Appendix*, Fig. S8), possibly contributing to strengthen a/c interaction as recently proposed for *E. coli* (21). Previous investigators have suggested that cardiolipin molecules stably bound in the vicinity of the a/c interface may play a role for functional rotation (24, 65). Our observations are consistent with this proposal, but our sampling timescales may not allow for the complete equilibration of the lipid distribution around the $F_{o}$ domain. Therefore, the presence of cardiolipin near the a/c interface may reflect in part the initial arrangement of the membrane rather than increased affinity of $F_{o}$ for cardiolipin. It has also been proposed that the central lumen of the c-ring in F- and V-ATPase is populated by lipids (66, 67), but their nature and stoichiometry is unknown in *Polytomella*. Whereas previous investigators modeled POPE (28, 68) or POPC (30, 31) in the lumen, we used cardiolipins, which may also contribute to explaining the different rotational PMFs. Future studies will have to address the interplay between lipids and the energetics of c-ring rotation.

## Conclusion

The functional, directional rotation of the c-ring is the starting point for the synthesis of ATP by ATP synthase. It is the result of a complex interplay between protonation/deprotonation, local dynamics of interactions, and large-scale subunit motion. We used atomistic free energy calculations to describe the structural mechanism and energetics of an elementary rotation step of the c-ring. Future studies will focus on integrating this structural description with a thermodynamically and kinetically consistent description of proton transfer to achieve a synthetic description of “osmo-mechanical” transduction by the $F_{o}$ rotor.

### Data Archival.

Simulation parameter files and all-atom structural models for representative configurations shown in Fig. 2 are accessible in the Zenodo repository http://10.5281/zenodo.8124466.

## Materials and Methods

### Preparation of Structural Models for Molecular Dynamics Simulation.

Models for states OOH and OHH were prepared from the *Polytomella sp.* ATP synthase cryo-EM structure (6RD9) by keeping the c-ring, a-subunit, and part of the peripheral stalk. Protonation states were determined by a multisite titration approach. Each model was embedded in a realistic IMM including cardiolipin, solvated in an orthorhombic box of TIP3P water molecules with 150mMNaCl, and energy-minimized under harmonic restraints. Then, NVT equilibration (T=300K) was run for 10ns followed by NPT equilibration (T=300K,P=1bar) for 20ns while harmonic restraints were progressively relaxed, except on the peripheral stalk. Details of the equilibration procedure and simulation parameters are given in *SI Appendix*.

### Molecular Dynamics Simulations.

MD simulations were run with GROMACS 2020.4 (69) using the CHARMM36m force-field (70). See *SI Appendix* for detailed parameters.

### Extended ABF Free Energy Calculations.

eABF calculations were run from the coordinates and velocities of the equilibrated structures using the *colvars* module (71) with GROMACS. Absolute harmonic restraints were applied on the CA atoms of the peripheral stalk; therefore, to avoid absolute-restraint-related artifacts, we ran these calculations in the NVT ensemble (T=300K). One eABF calculation was run per model (*i.e.,* one for OOH and one for OHH). Two-dimensional PMFs were computed along θ (*i.e.,* the rotation angle of the c-ring with respect to the cryo-EM structure, with structural alignment on the a-subunit) and $d_{1}$ (*i.e.,* the distance between cE111JCD and aR239CZ). To promote convergence of the calculations, an exploratory run was followed by stratified sampling (72). eABF simulations amount to a total of 68μs. One-dimensional PMFs along θ were obtained by Boltzmann-integration of the two-dimensional PMFs. Reweighting of eABF simulations was done as detailed in *SI Appendix*, using SciPy (73, 74) for interpolation and scikit-learn (75) for Kernel Density Estimation. See *SI Appendix* for details of the eABF protocol, convergence, and error analysis.

### Restrained-c-ring Simulations of State OOH with Protonated aE288.

64 configurations covering θ values ranging from ${-20}^{{}^{\circ}}$ to ${+30}^{{}^{\circ}}$ were extracted from OOH eABF simulations, and a proton was added on aE288. After minimization and equilibration, each configuration was used to run a restrained-c-ring simulation, *i.e.,* with a time-independent, $15\;\mathrm{kJ}/\mathrm{mol}/\deg^{2}$ harmonic restraint applied on θ. For comparison, identical simulations were also run without protonating aE288. Restrained-c-ring simulations amount to a total of 30μs. See *SI Appendix* for details.

### Water-Mediated Proton Transfer Analysis.

Water wires connecting residues aE288 and cE111A were identified by analyzing all stratified OOH simulations with the breadth-first algorithm implemented in MDAnalysis (76, 77). When applicable, the shortest water wire for a given frame was then identified using Dijkstra’s algorithm as implemented in NetworkX (78). The proton transfer score was defined as $k\left(\theta \right)=k_{0}\rho \left(\theta \right)\alpha^{{\left\langle n^{*}\right\rangle }_{\theta }}$ with $k_{0}=1$, ρ(θ) the occupancy (*i.e.,* existence probability) of at least one water wire of length ≤8 at c-ring rotational angle θ, ${\left\langle n^{*}\right\rangle }_{\theta }$ the average length of the shortest water wire at c-ring rotational angle θ (using a conditional average restricted to frames were at least one water wire is present) and α=0.8 an attenuation parameter, chosen to obtain appreciable signal. We derive the score and discuss the choice of α in *SI Appendix*. Restrained-c-ring simulations were analyzed in identical fashion. From these simulations, we further evaluated the typical water-bridge lifetime as the autocorrelation time of the water-bridge indicator function; see *SI Appendix* for details.

### Data Visualization and Rendering.

Molecular structures were visualized and rendered with VMD (79) and Pymol (80). Graphics were rendered with Matplotlib (81) and Inkscape (82).

## Supplementary Material

Appendix 01 (PDF)

## Data Availability

Molecular Dynamics setups and trajectories data have been deposited in Zenodo (http://doi.org/10.5281/zenodo.8124466) (83).
