# Supplementary material for: Mechanism of proton-powered c-ring rotation in a mitochondrial ATP synthase
Source: Proc Natl Acad Sci U S A. 2024 Mar 7;121(11):e2314199121. doi: 10.1073/pnas.2314199121 (PMC10945847; doi:10.1073/pnas.2314199121)
Supplement: Supplementary file 1 — Appendix 01 (PDF) [file pnas.2314199121.sapp.pdf]

Supporting Information for

Mechanism of proton-powered c-ring rotation in a mitochondrial ATP synthase

Florian E. C. Blanc and Gerhard Hummer[1]

[1]To whom correspondence may be addressed. E-mail: gerhard.hummer@biophys.mpg.de

This PDF file includes:

Supporting text
Figs. S1 to S10
Tables S1 to S5
SI References

Supporting Information Text

Detailed Methods

Atomistic models of ATP synthase F_o region in a realistic Inner Mitochondrial Membrane.

Protein model. To study the rotation of the c-ring, we built a minimal model of the F_o region by extracting the c-ring, the a-subunit and part of the peripheral stalk from the recently published 2.8 Å-resolution cryo-EM structure of *Polytomella sp.* ATP synthase (1) (PDB:6RD9), see Main Text Figure 1b and Table S1. The missing loop of the a-subunit (residues 205 to 219) was reconstructed with MODELLER (2) using available lower-resolution coordinates as a template (3). The missing C-terminal residues of the a-subunit were built *de novo* with MODELLER. The electronic density near aHis248 was modelled as a single Zn^{2+} ion.

Protonation states. Protonation states were assigned according to the following protocol. First, residues cE111A and cE111J were assigned the standard, negatively charged state (*i.e.,* the system was modelled in state OOH, see Table S5). Then, the most probable protonation states as a function of pH for all other titratable residues (ASP, GLU, LYS, HIS) were determined by multisite titration pK_A calculations (4). Following standard practice, ARG residues were all modelled in the positively charged form. This is consistent with recent findings that arginine is virtually never deprotonated in proteins, even in hydrophobic environments (5). The protein interior, solvent and membrane were treated as continua of dielectric constants 4.0, 80, and 4.0 respectively. The membrane was modelled as a 3.5 nm-thick slab. Ions in the solvent were modelled by a Boltzmann-distributed charge density corresponding to 150 mM NaCl at 300 K. APBS (6) with the tAPBS front-end was used to solve the Poisson-Boltzmann equation for the electrostatic potential. Then, protonation probabilities as a function of pH were evaluated by Monte Carlo sampling with the Karlsberg2 program (7, 8). When appropriate, solvent-exposed residues were assigned the protonation state corresponding to the pH of their membrane compartment, *i.e.,* $pH = 6.8$ for the IMS and $pH = 7.7$ for the matrix. Notably, this resulted in residue aE225 being protonated and residue aE288 being deprotonated. For all titratable residues on c-ring subunits (except cE111) a consensus protonation state was determined by the majority rule. To model state OHH, we added a proton on cE111A, but did not perform a new pK_A calculation, that is, we neglect the possible impact of changes in protonation of cE111A on other titratable residues. This ensures that the protonation state of cE111A is the only changing parameter between models. Structural models were prepared for MD simulation using CHARMM.

Membrane model. A membrane patch with average composition of cauliflower inner mitochondrial membrane (9) (see Table S1) was built using CHARMM-GUI (10–12) and equilibrated by 20 ns of molecular dynamics with GROMACS. Cardiolipin (CDL) molecules were modelled in the CDL^{2-} state. The protein was inserted in the membrane by deleting overlapping lipids. Cardiolipin molecules in the central hole of the c-ring were kept; clashes between them and the c-ring atoms were relieved by energy-minimizing them in a cylindrical harmonic potential centered on the c-ring axis (using the MMFP module of CHARMM).

Preparation for molecular dynamics simulation. The system was embedded in an orthorhombic box of dimensions 15.1 nm × 12.5 nm × 17.5 nm and solvated with TIP3P water molecules. Na^+ and Cl^- ions were added to ensure electroneutrality of the system and achieve an approximate salt concentration of 150 mM. GROMACS-format topology files were obtained using the psf2itp.py program from the CHARMM-GUI suite (13). Each system was energy-minimized with absolute harmonic restraints on the protein CA atoms, the Zn^{2+} ion, and lipid head-groups (force constant 4184 kJ/mol/nm^2).

Molecular dynamics simulations.

General parameters. The energetics was modelled with the CHARMM36m force-field (14) with a van-der-Waals cutoff of 1.2 nm and using van-der-Waals force-switching from 1 nm. Electrostatics were treated by the Particle Mesh Ewald method for the long-range component, and the cutoff between short-range and long-range was set to 1.2 nm. The Stote-Karplus parameters were used for the Zn^{2+} ion (15).

All molecular dynamics simulations were performed with GROMACS 2020.4 (16) using the leap-frog integrator with a 2 fs timestep. Covalent bonds involving hydrogens were constrained using LINCS. In all production simulations, the peripheral stalk residues were restrained close to their initial positions using absolute harmonic restraints on CA-atoms (force constant 4184 kJ/mol/nm^2). Temperature was maintained at 300 K using the velocity-rescale thermostat (17) with a coupling time of 100 ps on three separate temperature groups (protein atoms, membrane atoms, water and ions). During NPT equilibration, pressure was maintained at 1 bar using a semi-isotropic Berendsen barostat (xy and z) with a time constant of 1 ps and compressibility 4.5×10^{-5} bar^{-1} in both directions.

Equilibration protocol. After energy minimisation, each model was equilibrated according to the following 4-step protocol designed to progressively relax the membrane and protein.

1. 2 ns of NVT equilibration ($T = 300$ K, Berendsen thermostat) with harmonic restraints on the protein backbone heavy atoms and the heavy atoms of all lipid head groups.

2. 4 ns of NVT equilibration ($T = 300$ K, Berendsen thermostat) with harmonic restraints on the protein backbone heavy atoms, the Zn^{2+} ion and the heavy atoms of the z-coordinate of all lipid head groups.

3. 4 ns of NVT equilibration ($T = 300$ K, Berendsen thermostat) with harmonic restraints on the protein backbone heavy atoms and the Zn^{2+} ion, but all lipid restraints removed.

4. 20 ns of NPT equilibration ($T = 300$ K, Berendsen thermostat, $P = 1$ bar, Berendsen barostat) with harmonic restraints on the peripheral stalk backbone heavy atoms.

Extended Adaptive Biasing Force (eABF) calculations.

Simulation setup eABF calculations were performed to explore functional c-ring rotation. The use of an adaptive biasing force (ABF) enhances the sampling along two Collective Variables (CVs) by applying a biasing force. This biasing force is adaptively updated to compensate for the generalized thermodynamic force felt by the CVs. Thus, at convergence biased CVs undergo purely diffusive dynamics. At any time, numerical integration of the collected biasing force enables the estimation of the free energy landscape. Here, 2D integration was performed by Poisson integration as described in reference (18). In eABF, ABF dynamics is applied on a virtual degree of freedom harmonically coupled to the CV (19). Two-dimensional eABF calculations were run in NVT conditions starting from the temperature- and pressure-equilibrated systems, using the colvars module (20) interfaced with Gromacs 2020.4 and with active positional restraints. The NVT ensemble was chosen to avoid artefacts due to the usage of absolute positional restraints. To limit non-equilibrium effects, the *fullSamples* parameter was set to 10 000; that is, the bias is not applied at a given point of configurational space until this point has been visited at least 10 000 times to ensure a robust initial estimate of the bias. eABF was applied on θ, *i.e.,* the c-ring rotation angle with respect to its initial position, and on d_1J (called d_1 in Main Text), *i.e.,* the separation distance between cE111J CD and aR239 CZ. θ was defined using an orientation quaternion (spinAngle collective variable) computed on the CA atoms of residues (55 58 61 64 67 70 73 76 79 82 85 88 91 95 98 101 104 107 110 113 116 119 122 125) for each c-ring subunit, and using the Z-axis $(0, 0, 1)$ as rotation axis (20). The calculations were performed on a rectangular grid, with θ between -47° and 41° in 0.5° increments, and $d_1J \in 25$ nm to 2.5 nm in 0.025 nm increments. In this way, we covered two full 36° rotation steps, one in the forward direction and the other in the reverse direction. Both CVs were harmonically coupled to extended degrees of freedom undergoing Langevin dynamics at temperature T =300 K, using force constants of 10 kcal/mol/deg^2 for θ and 10 kcal/mol/Å^2 for d_1J. The Corrected z-averaged restraint (CZAR) estimator was used to correct free energy gradient estimates (19). To keep the c-ring aligned with the Z-axis, we applied harmonic restraints on two virtual atoms, respectively close to the c-ring central axis' top and bottom. Specifically, the centers of geometry of the CA atoms of residues (90 96) for all c-ring subunits, and of CA atoms of residue 59 for all c-ring subunits, were harmonically restrained (force constant 10 kcal/mol/Å^2) to their respective position in the equilibrated structure. This mimics the presence of the central stalk in full-length ATP synthase. The eABF bias was occasionally observed to induce irreversible local unfolding of α-helices. When this happened, the simulation was backtracked until slightly before unfolding and relaunched with harmonic walls on the backbone inter-atomic distances. The harmonic wall potential $U_{wall}(d)$ applied on a backbone O−N distance d is given in equation 1.

$$U_{wall}(d) = \begin{cases} \frac{1}{2}k\left(d - (\bar{D} + 1.1\,\text{Å})\right)^2 & \text{if } d > \bar{D} + 1.1\,\text{Å} \\ 0 \text{ otherwise.} \end{cases} \quad [1]$$

In equation 1, $k = 20$ kcal/mol/Å^2 and $\bar{D}$ is the average distance during the first 50 ns of eABF simulation. Harmonic walls preserve local helix flexibility while preventing dramatic, irreversible unfolding. To promote convergence of eABF sampling, we used the two-step strategy introduced in reference (21). After an initial long exploratory eABF run, CV-space was divided into non-overlapping windows and an eABF simulation was launched in each window starting from configurations sampled in the exploratory run, with the eABF bias accumulated in the exploratory run. Specifics of domain definition (Supplementary Figure S1c and d) and sampling times (Supplementary Table S2) differ slightly between states OOH and OHH.

Convergence and error analyses Convergence of the eABF calculations was evaluated by monitoring the coverage of CV-space (Supplementary Figure S1c and d) and the Root Mean Square Deviation (RMSD) of the eABF force estimate (*i.e.,* the estimate of the free energy gradient) (Supplementary Figure S2). The RMSD was computed separately for the uncorrected and CZAR-corrected gradient estimates. Most windows exhibit a stable gradient-RMSD in the last ≈100 ns of stratified eABF simulation, indicating that the bias has reasonably converged. The statistical error was estimated using a bootstrapping procedure based on the standard error of the mean of the accumulated biasing force, see Supplementary Figure S3 (21, 22). We note that this procedure yields the error associated to the uncorrected potential of mean force rather than the CZAR-corrected ones. In absence of a rigorous procedure to propagate statistical errors through the CZAR-estimator, we chose to use the uncorrected errors to quantify uncertainty on the CZAR-corrected profiles. Estimated statistical errors do not exceed 0.2 kcal mol^{-1}, indicating a low variance of the force-estimate which, together with the coverage of the relevant regions of configurational space (Supplementary Figure S1c and d), suggests proper convergence of the eABF calculation.

Reweighting of eABF calculations. Estimating the free energy landscape along degrees of freedom other than θ and d_1J requires reweighting to correct for the eABF bias. Reweighting of eABF calculations is challenging because of the non-stationary bias. To our knowledge, there exists no rigorous procedure for this purpose. Nevertheless, approximate reweighting of orthogonal degrees of freedom can be achieved using the final, converged PMF to compute unbiasing factors - similar to an approach previously used for metadynamics (23). With $A_\infty(\theta, d_1J)$ the converged free energy profile from ABF, the applied biasing potential is $-A_\infty$. Thus, the (approximately) unbiased weight of configuration x_i from the eABF trajectory is:

$$w(x_i) = e^{+\beta A_\infty(\theta(x_i), d_1 J(x_i))} \quad [2]$$

where $\beta = 1/k_B T$. If configuration x_i has unbiased probability $P(x_i)$ (*e.g.* in a long, unbiased equilibrium trajectory) and probability $\tilde{P}(x_i)$ in the (converged) ABF simulation, then:

$$P(x_i) \propto e^{-\beta A_\infty(\theta(x_i), d_1 J(x_i))} \tilde{P}(x_i) \quad [3]$$

We implemented this reweighting procedure as follows. First, a bivariate spline is fitted to the PMF estimate, which makes it possible to compute the reweighting factor for any point in $(\theta, d_1 J)$ space. Spline fitting is performed using the RectBivariateSpline function of SciPy (24, 25). Second, this spline approximation is used to compute reweighting factors (equation 2) which are then used in a weighted Kernel Density Estimation function, as implemented in scikit-learn (26). For the reweighted PMF shown in Main Text Figure 3a, we used Gaussian kernels of bandwidth 0.1 nm for the donor-acceptor distance, and 1° for the c-ring rotation angle θ. Reweighting factors can also be used in weighted average calculations to unbias canonical averages, as is done in Main Text.

Restrained-c-ring simulations of state OOH with protonated aE288. To assess the importance of the protonation state of aE288, we added a proton to aE288 on conformers extracted from the state OOH eABF simulation, and re-sampled them using conventional MD with a time-independent harmonic restraint on θ. Configurations covering θ values ranging from -20° to 30° were selected along the approximate minimal free energy path on the 2D free energy surface from Supplementary Figure S1a. For each conformer, a proton was added on aE288 using the CHARMM GLUP patch. Then, each system was energy-minimized and equilibrated with the same protocol as to prepare eABF calculations. Finally, NVT molecular dynamics simulation with the same general parameters as for eABF was run, except that the eABF bias was replaced by a time-independent harmonic restraint on θ of force-constant 15 kJ/mol/deg^2. To make comparison with unprotonated aE288 simulations less contingent on simulation protocols, we also re-sampled these conformers using the same protocol but leaving aE288 unprotonated. Corresponding simulation times are reported in Table S3.

Water bridge analysis. Water bridge analysis was performed using the dedicated module in MDAnalysis version 2.0.0 (27, 28). In brief, this module uses a breadth-first algorithm to iteratively search for water-mediated hydrogen bond bridges between selected residues. The length of shortest water bridge was computed with Dijkstra's algorithm in networkx (29).

Derivation of the proton transfer score. We define an effective proton transfer score to evaluate the proton transfer efficiency as a function of the rotational state of the c-ring. Inspired by reference (30), we reasoned that the proton transfer requires either a direct hydrogen-bond connection or the existence of at least one bridging water wire, and that proton transfer efficiency attenuates geometrically with the number of water molecules in the wire. For a given donor/acceptor/water configuration x_i (*i.e.,* the Cartesian coordinates of the system, *e.g.,* a simulation frame), this corresponds to the proton transfer efficiency model given in equation 4.

$$k(x_i) = k_0 \sum_{n \geq 1} \Omega_n(x_i) \alpha^n \quad [4]$$

In equation 4, k_0 is a basal efficiency, $\Omega_n(x_i)$ is the number of distinct water wires of length n at configuration x_i, and $\alpha < 1$ is a dimensionless attenuation factor quantifying the decrease in proton transmission probability as a new water molecule is added to the wire. Note that direct proton transfer could also be taken into account by extending the sum to $n = 0$ and setting $\Omega_0 = 1$ if direct proton transfer is possible, 0 otherwise. Here, we focus on water-mediated proton transfer and thus exclude the $n = 0$ term from the sum. Because of the geometric attenuation, the sum in equation 4 will be dominated by $n^*(x_i)$, *i.e.,* the smallest n for which $\Omega_n(x_i) \neq 0$. We thus use the approximation

$$k(\theta) = k_0 \rho(\theta) \alpha^{\langle n^* \rangle_\theta} \quad [5]$$

where $\rho(\theta) \equiv \langle \mathbb{I}_{n \geq 1}(x_i) \rangle_\theta$ is the water-wire density, with $\mathbb{I}_{n \geq 1}(x_i)$ an indicator function taking value 1 if at least one water wire of length $n \geq 1$ exists in configuration x_i. The conditional averages $\langle \cdots \rangle_\theta$ are over all frames x_i with given values of θ. The values of $\langle n^*(x_i) \rangle_\theta$ were determined from eABF simulations in state OOH (with or without eABF-reweighting), as shown in Supplementary Figure S6b, and from c-ring-restrained simulations of state OOH with aE288 charged or protonated, as shown in Supplementary Figure S7d.

This formulation allows us to use directly the observables $\rho(\theta)$ and $\langle n^* \rangle_\theta$ from eABF simulations, taking $k_0 = 1$ since we are not concerned with the precise value of the score. Main Text Figure 3d shows the score computed from eABF simulations of state OOH (with or without eABF-reweighting) using $\alpha = 0.8$, chosen to obtain appreciable signal. Supplementary Figure S7e shows the score computed from c-ring-restrained simulations using $\alpha = 0.8$. Supplementary Figure S6c illustrates the influence of α on the score profile. α is expected to depend in complex fashion on the translational and re-orientational dynamics of water molecules within a wire (30, 31).

Since our study focuses on comparing relative proton transfer efficiency to identify proton-transferring states, the approximations are appropriate, because they are not expected to dramatically change the qualitative picture. In the future, usage of the full expression for the score (*i.e.,* an averaged version of equation 4) and a careful parametrization of k_0 and α, for example from *ab initio* Molecular Dynamics simulations, could enable quantitative investigations of proton transfers in ATP synthase and other systems.

Lifetime of water bridges. Because c-ring-restrained simulations use a time-independent bias to keep θ close to a fixed value, they are more appropriate to evaluate water bridge lifetime as a function of θ than eABF simulations, in which θ is allowed to evolve dynamically. From the c-ring-restrained simulations, the lifetime of water bridges was evaluated as the autocorrelation time of the water bridge indicator function $\mathbb{I}_{n\geq 1}$ computed over individual trajectories of c-ring-restrained simulations (with one frame saved every 100 ps). First, the (normalized) autocorrelation function $C(\tau)$, defined in equation 6, was estimated from the trajectory using the np.convolve function from Numpy.

$$C(\tau) = \frac{1}{M} \lim_{t\to+\infty} \frac{1}{t} \int_0^t \mathbb{I}_{n\geq 1}(x(t'))\mathbb{I}_{n\geq 1}(x(t'+\tau))\mathrm{d}t', \; M = \langle \mathbb{I}_{n\geq 1}(x(t))^2 \rangle \quad [6]$$

Then, a single decaying exponential was fitted to $C(\tau)$ with SciPy, see equation 7.

$$C(\tau) \underset{\text{fit}}{=} \exp\left(-\frac{\tau}{\tau_{WB}}\right) \quad [7]$$

The autocorrelation time τ_{WB}, which is the sole adjustable parameter, is identified with the lifetime of the water bridge. Lifetime values are reported on Supplementary Figure S7f.

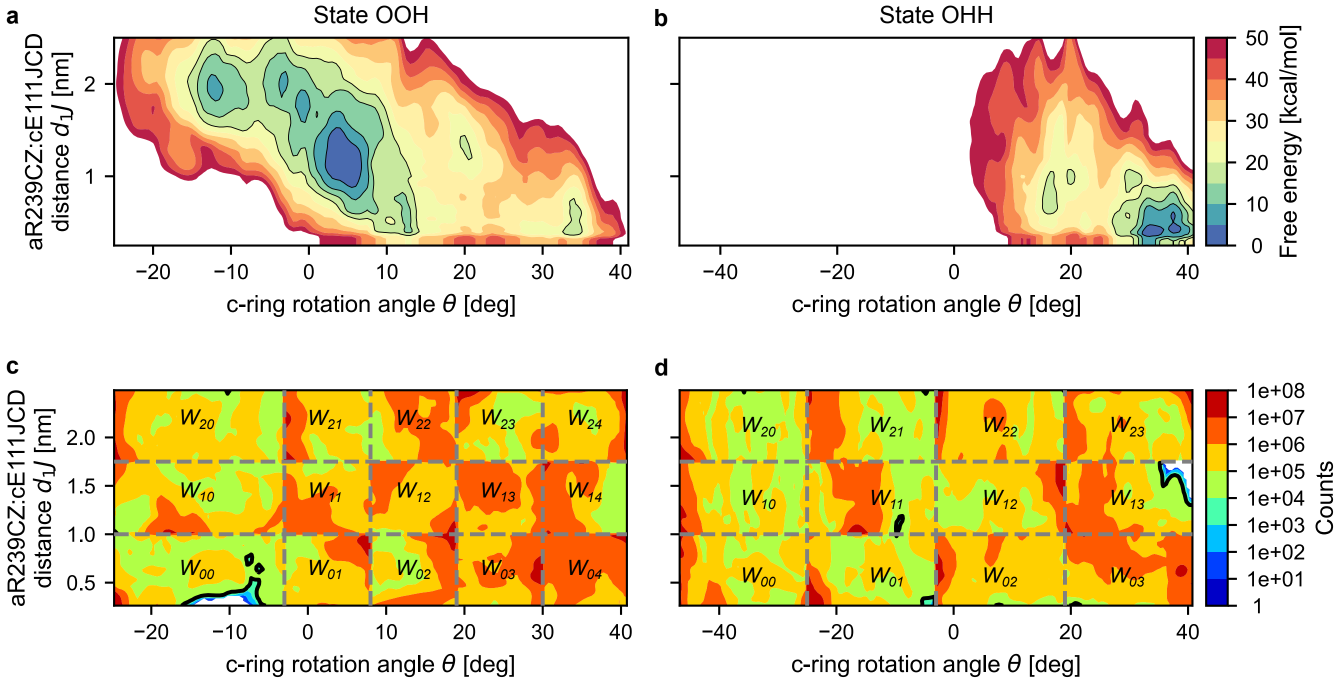

Fig. S1. 2D free energy landscapes computed by eABF. (a) Pre-protonation state (OOH). (b) Post-protonation state (OHH). (c-d). Sampling of the configurational space by eABF simulations. Shown are the respective counts in the 2D bins.

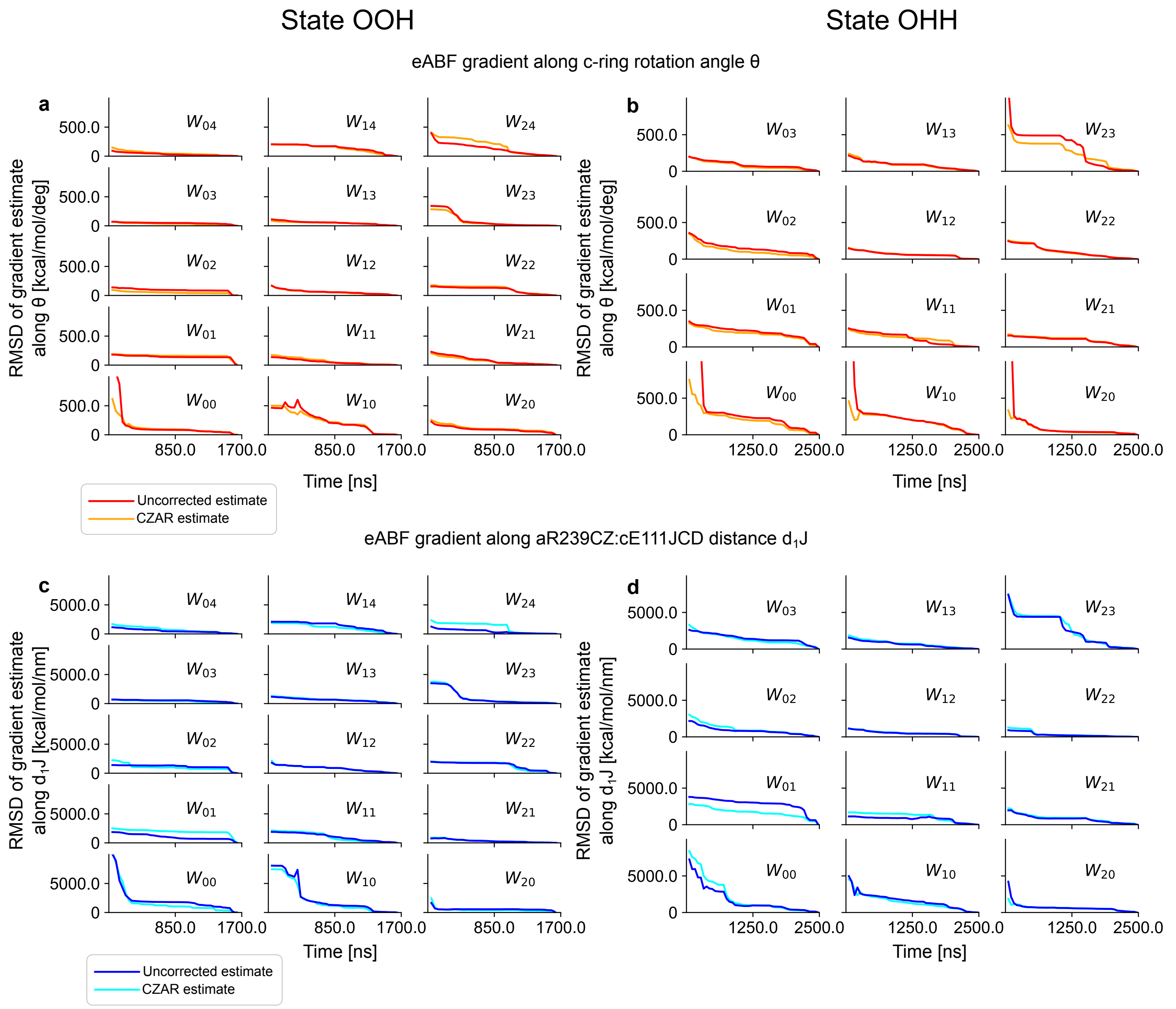

Fig. S2. Convergence of eABF force estimate in stratified eABF simulations. In most windows, the gradient-RMSDs have stabilized in the last $\approx 100\,\mathrm{ns}$, indicating that the eABF bias is reasonably converged.

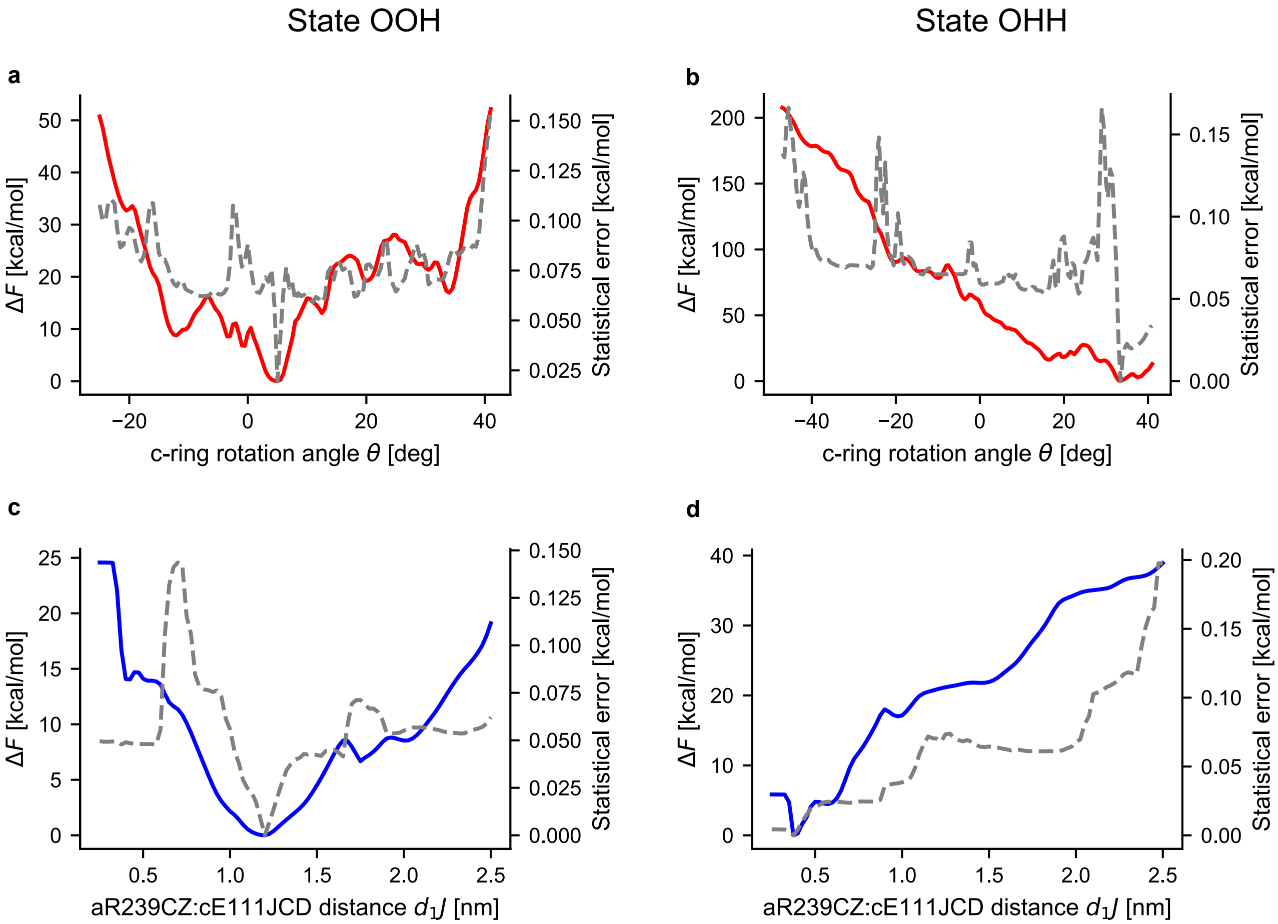

Fig. S3. Bootstrap error analysis of eABF simulations. In panels (a) to (d), the free energies (left scale) are shown as solid colored lines, and the statistical errors of the PMF estimated by bootstrap are shown as dotted grey lines.

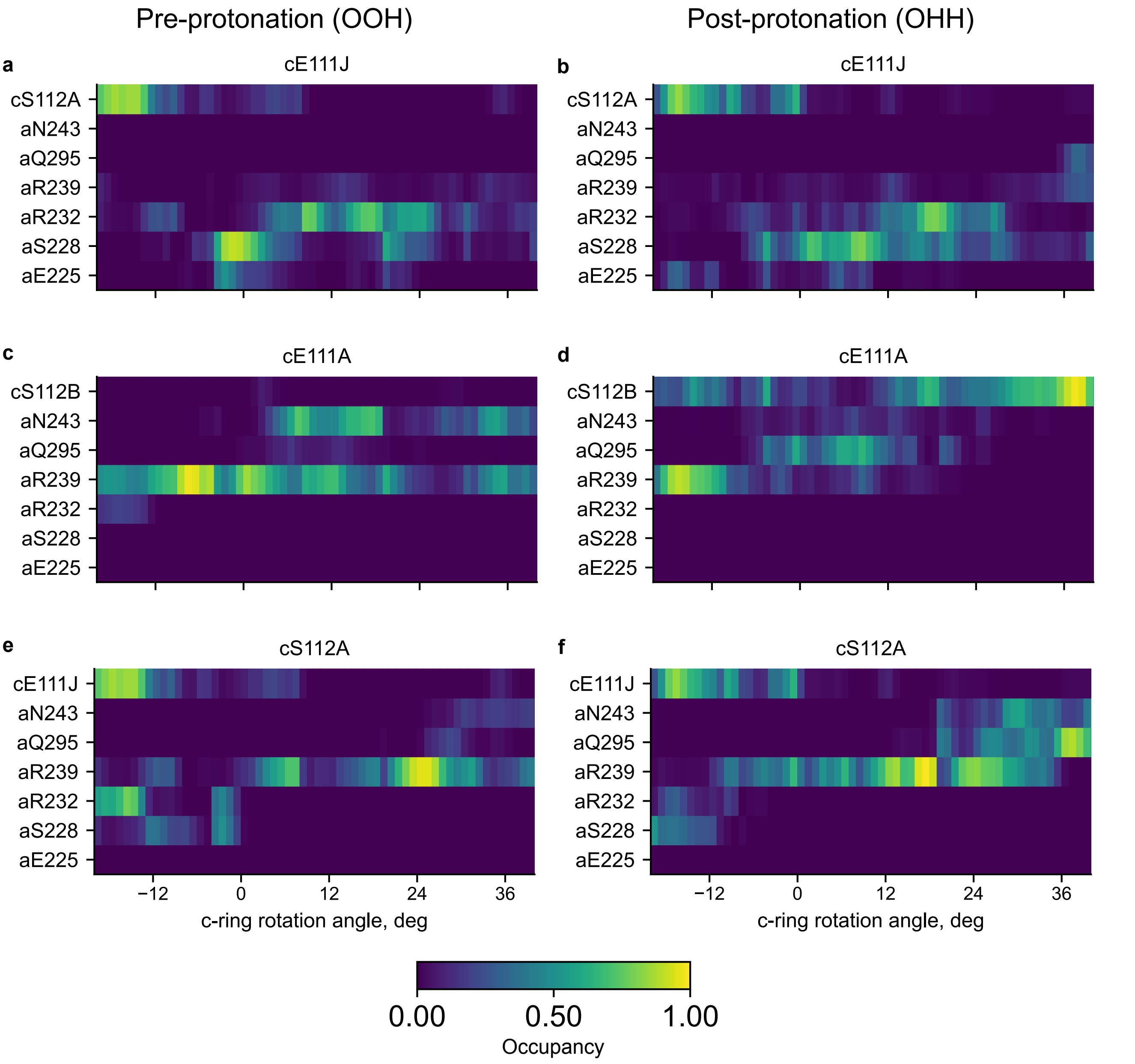

Fig. S4. Contact map analysis of the polar interactions between c-ring and a-subunit in eABF simulations. The color indicates the probability of a particular contact to be formed at a given c-ring rotation angle (scale at bottom). Note that the weak signals observed along the aR239 row in panels a and b come from the corresponding aR239:cE111J distance being biased in the eABF calculation. At convergence, uniform sampling is expected in the interval $[0.25\,\mathrm{nm}, 2.5\,\mathrm{nm}]$. For the contact threshold of $0.5\,\mathrm{nm}$ used here, this translates into a predicted $(0.5-0.25)/(2.5-0.25) \simeq 11\%$ contact probability, which is consistent with the observed values.

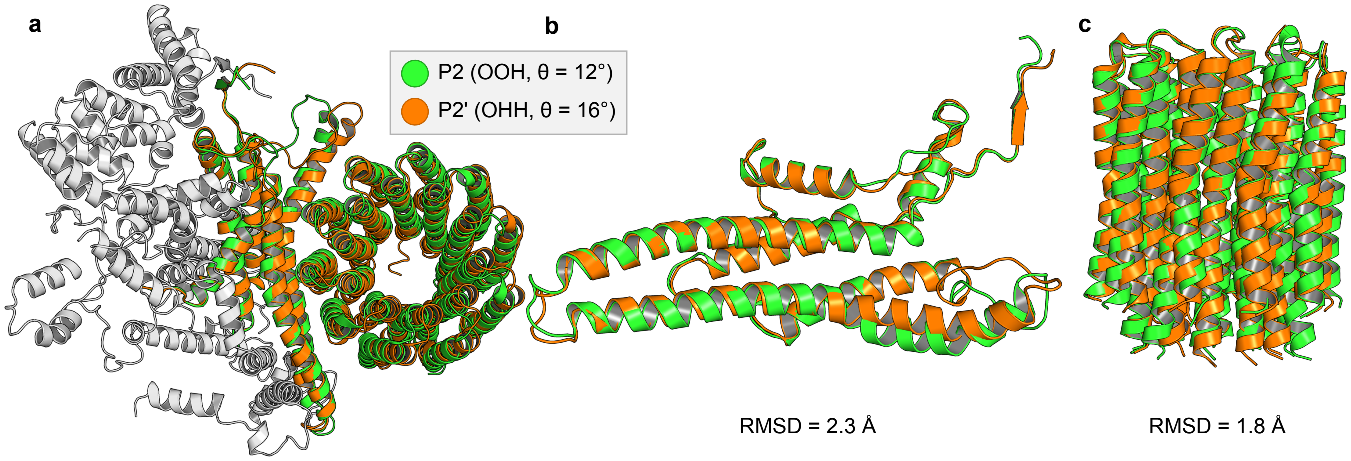

Fig. S5. Comparison of P2 and P2' representative structures. (a) Overview. (b) a-subunit. (c) c-ring. The comparison reveals that protonation of the rotor does not create major conformational changes in the a-subunit and c-ring besides the 4° rotation.

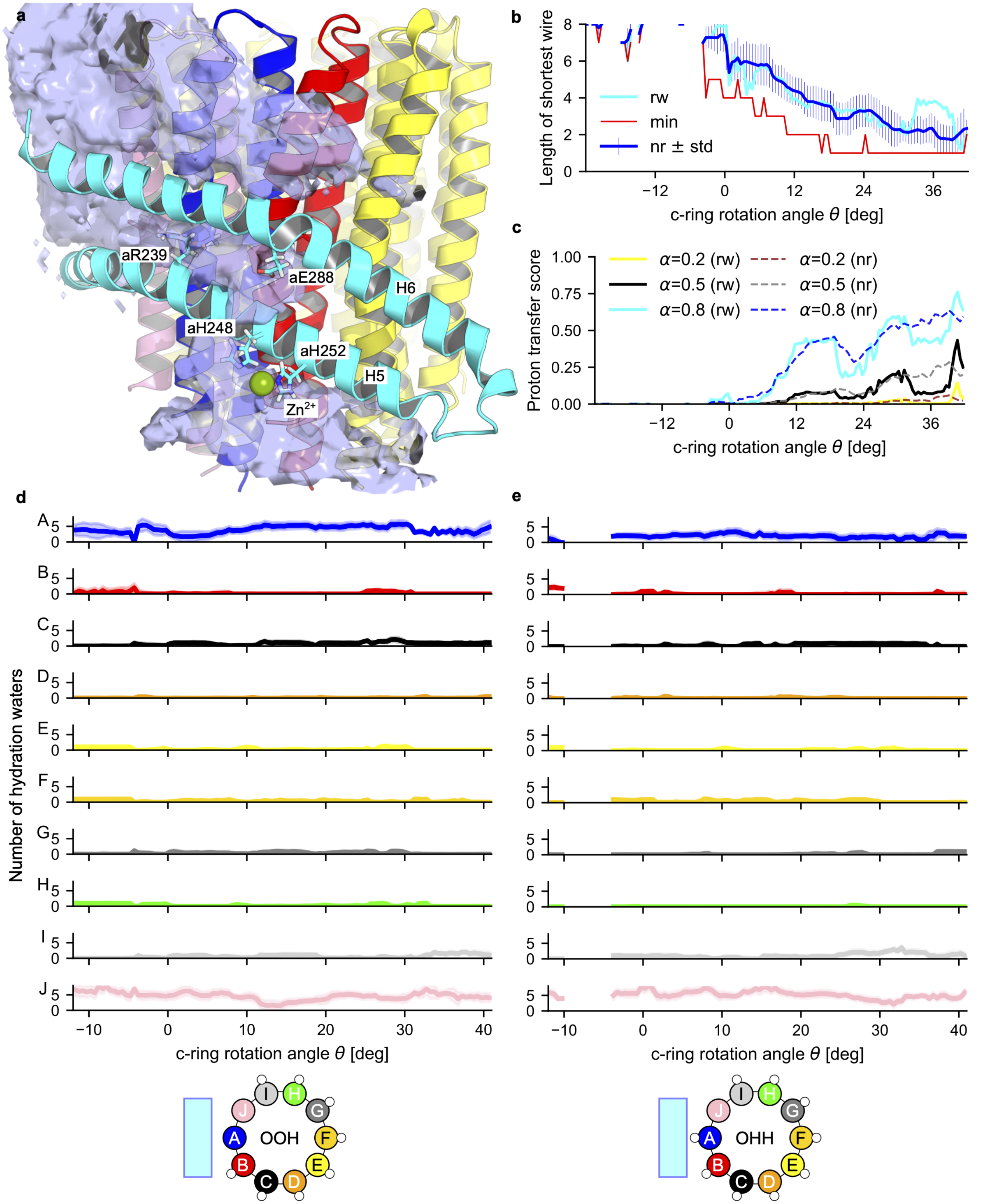

Fig. S6. Water-chain analysis in eABF simulations. (a) Water populates the a/c interface through the access and exit half-channels. Shown is the water density averaged over the first $45\,\mathrm{ns}$ of stratified eABF simulation in state OOH and window W_{11}, which is representative of the water distribution in the pre-rotation configuration. For clarity, only helices H5 and H6 of the a-subunit are displayed. Water enters the access half-channel through an opening between H5 and H6 in the vicinity of aH248, aH252, the Zn^{2+} ion and aE288. (b) θ-dependent length of the shortest water bridge (conditioned on existence). Shown are the eABF-reweighted average (cyan), the minimal sampled value (red) and the non-reweighted average $\pm$ standard deviation. (c,d) θ-dependent average number of hydration waters for all 10 cE111 side chains in state OOH (c) and OHH (d). Shown are the eABF-reweighted averages $\pm$ standard deviation. Sketches at the bottom illustrate protonation states and coloring and labelling conventions. Hydration waters are defined as within $3.4\,\text{Å}$ of the carboxylate oxygens.

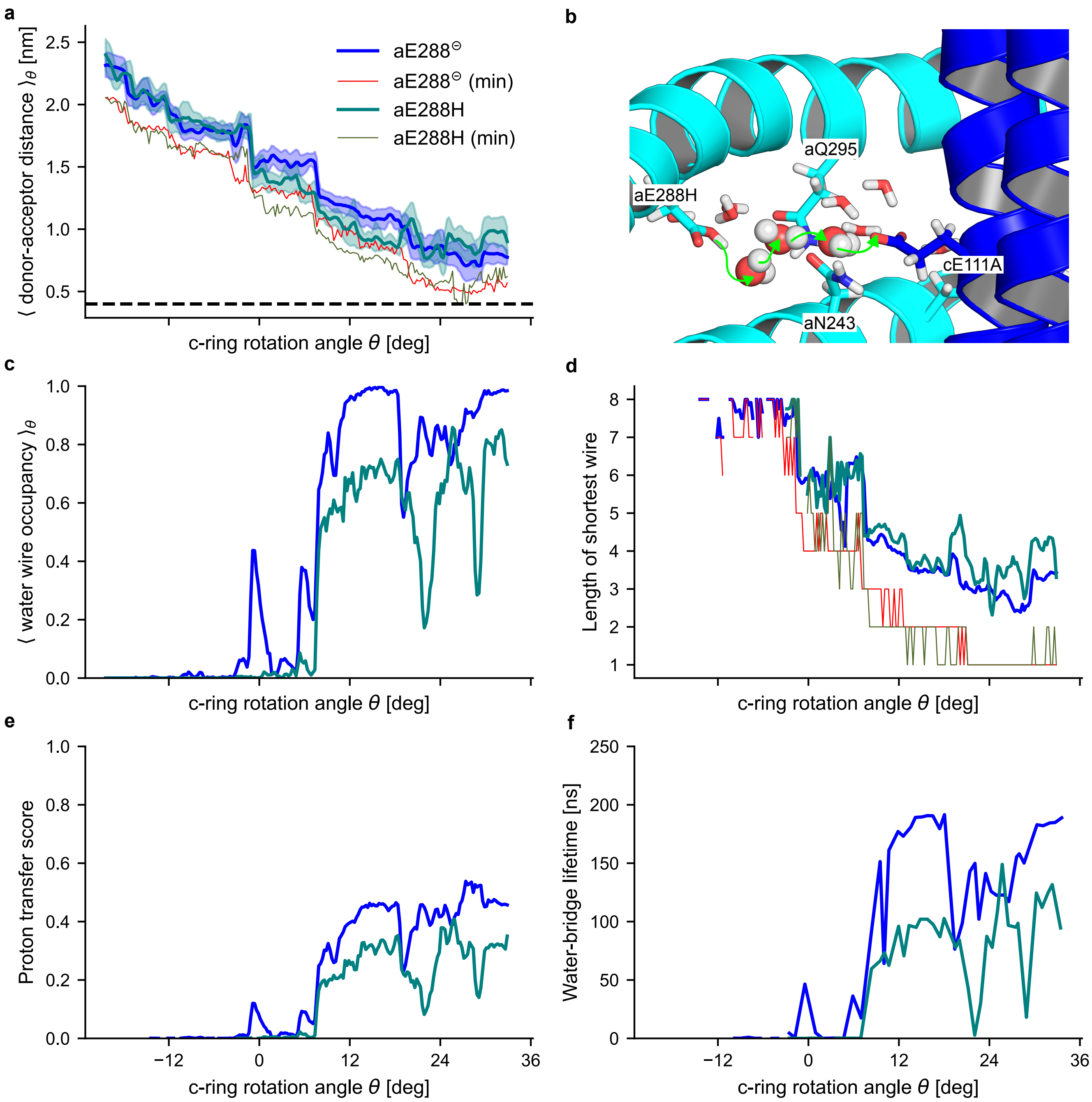

Fig. S7. Effect of aE288 protonation on water-mediated proton transfer from c-ring-restrained simulations. (a) Donor (aE288) - acceptor (cE111) distance as a function of θ when aE288 is charged or protonated. Shown are the θ-conditional averages (thick lines) $\pm$ standard-deviation (shading) and the minimal sampled values for a given θ (thin lines). The horizontal dashed line indicates the distance where a direct proton transfer would be feasible. (b) Representative bridge of 3 water molecules between protonated aE288 (aE288H) and cE111A in state P2 captured in c-ring restrained simulations. (c) θ-dependent occupancy of water wires of length ≤ 8. (d) θ-dependent length of the shortest water bridge (conditioned on existence). Shown are the averaged (thick lines) and minimal (thin lines) values. (c) Water-mediated proton transfer score as a function of θ. (d) θ-dependent water-bridge lifetime, estimated as the autocorrelation time of the indicator function of water bridge existence. Overall, the analyses shown in this figure indicate that protonation of aE288 slightly decreases the stability of water bridges and the efficiency of proton transfer, but does not otherwise affect our conclusions that proton-transfer is maximally probable in the vicinity of state P2 ($\theta \approx 12^\circ$)

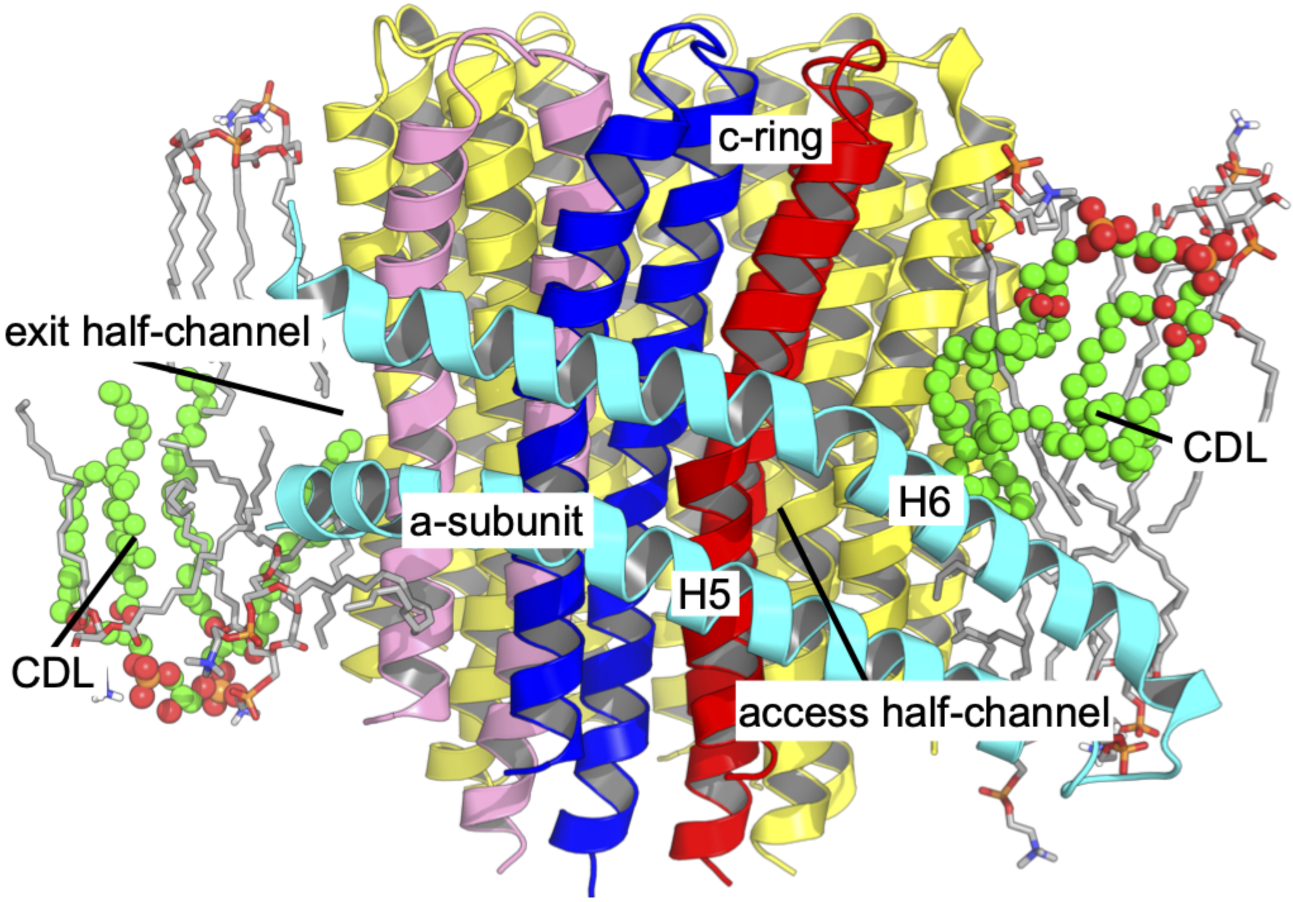

Fig. S8. Lipid molecules in the vicinity of the a/c interface. Shown is the equilibrated structure of state OOH as an example of typical lipid distribution. POPE/POPC/POPI (grey sticks) and cardiolipin molecules (CDL, green spheres) mediate a/c contacts.

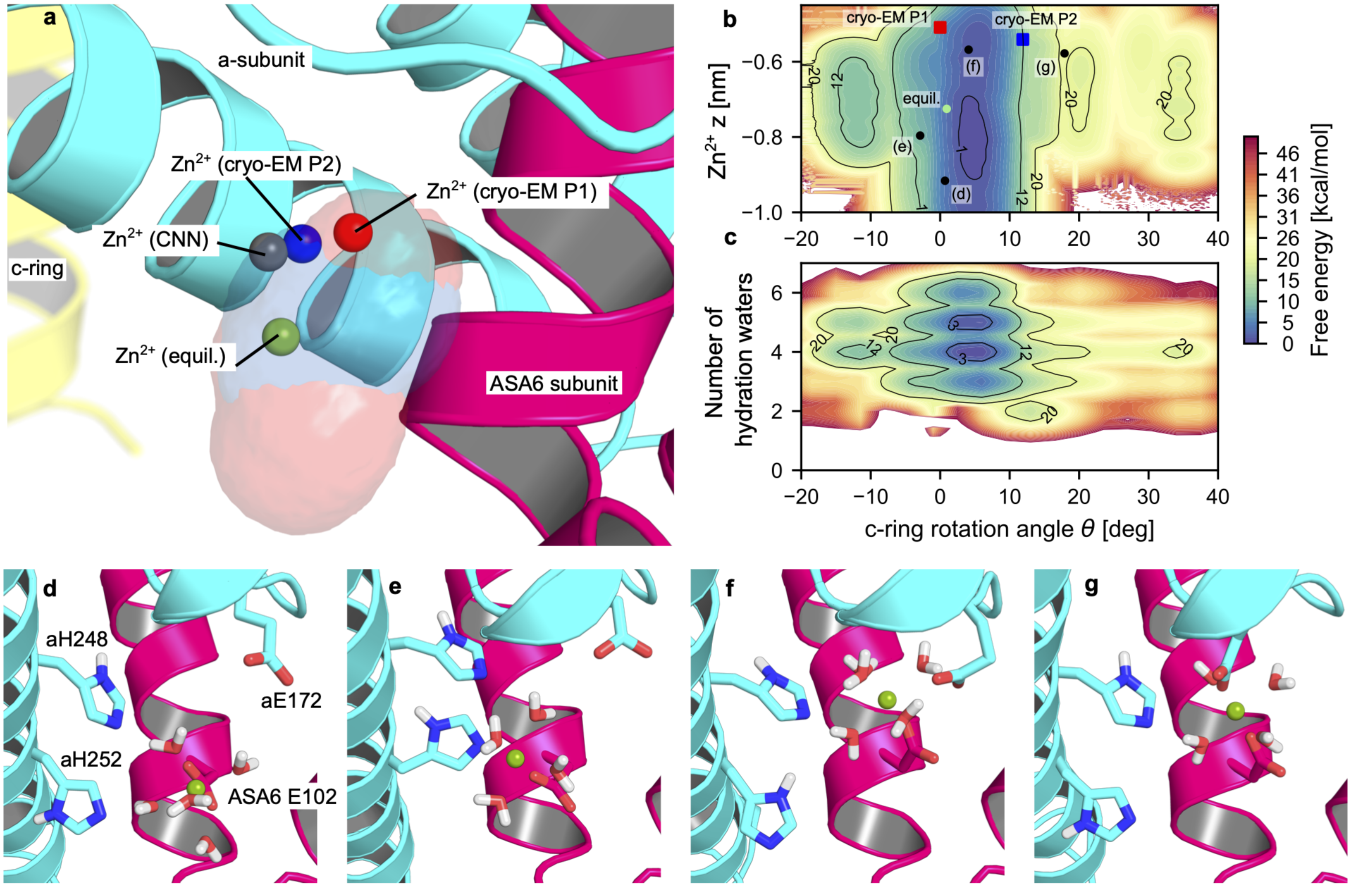

Fig. S9. Dynamics of the a-subunit-bound Zn^{2+} ion. (a) Alternate positions explored by Zn^{2+} in cryo-EM and eABF simulations. Shown are the state OOH equilibrated structure, and the Zn^{2+} ion in cryo-EM P1 (red sphere), cryo-EM P2 (blue sphere), convolutional neural network prediction (grey sphere) and state OOH equilibrated structure (green sphere). Also shown are the $0.01\,\text{Å}^{-3}$ isocontours of the Zn^{2+} positional probability densities collected by averaging (without reweighting) over the eABF windows W_{11} (*i.e.,* , representative of the P1 basin, transparent red surface) and W_{12} (*i.e.,* , representative of the P2 basin, transparent blue surface). (b) eABF-reweighted free energy landscape of Zn^{2+} z-coordinate (relative to the center of geometry of the ASA6 subunit) versus θ. Configurations shown in a. and (d-g) are positioned on the plot. (c). eABF-reweighted free energy landscape of the number of water molecules in the first hydration shell of Zn^{2+} (*i.e.,* within $3\,\text{Å}$) versus θ. Gaussian KDE with bandwidth = 0.1 is used to turn discrete hydration numbers into continuous values. Taken together, (b) and (c) show that Zn^{2+} undergoes extensive fluctuations of position (roughly $5\,\text{Å}$ of amplitude) and hydration in the OOH ground state. The transition to P2 restricts these fluctuations. (d-g) Typical simulation frames illustrating the alternate positions and hydration patterns of Zn^{2+}. Notably, we observe that 1) Zn^{2+} is always interacting directly with residue ASA6 E102, and through a water-mediated interaction with aH248, that 2) downward poses (d-e, $z < -0.8\,\text{nm}$) entail interactions (either direct or water-mediated) with aH252, but not aE172, whereas 3) the opposite is true for upward poses (f-g, $z > 0.8\,\text{nm}$). This indicates that the positional dynamics of Zn^{2+} is controlled by competition between aE172 and aH252 for interaction, and that interaction with aE172 is favored when the c-ring is in state P2 through a yet unknown mechanism.

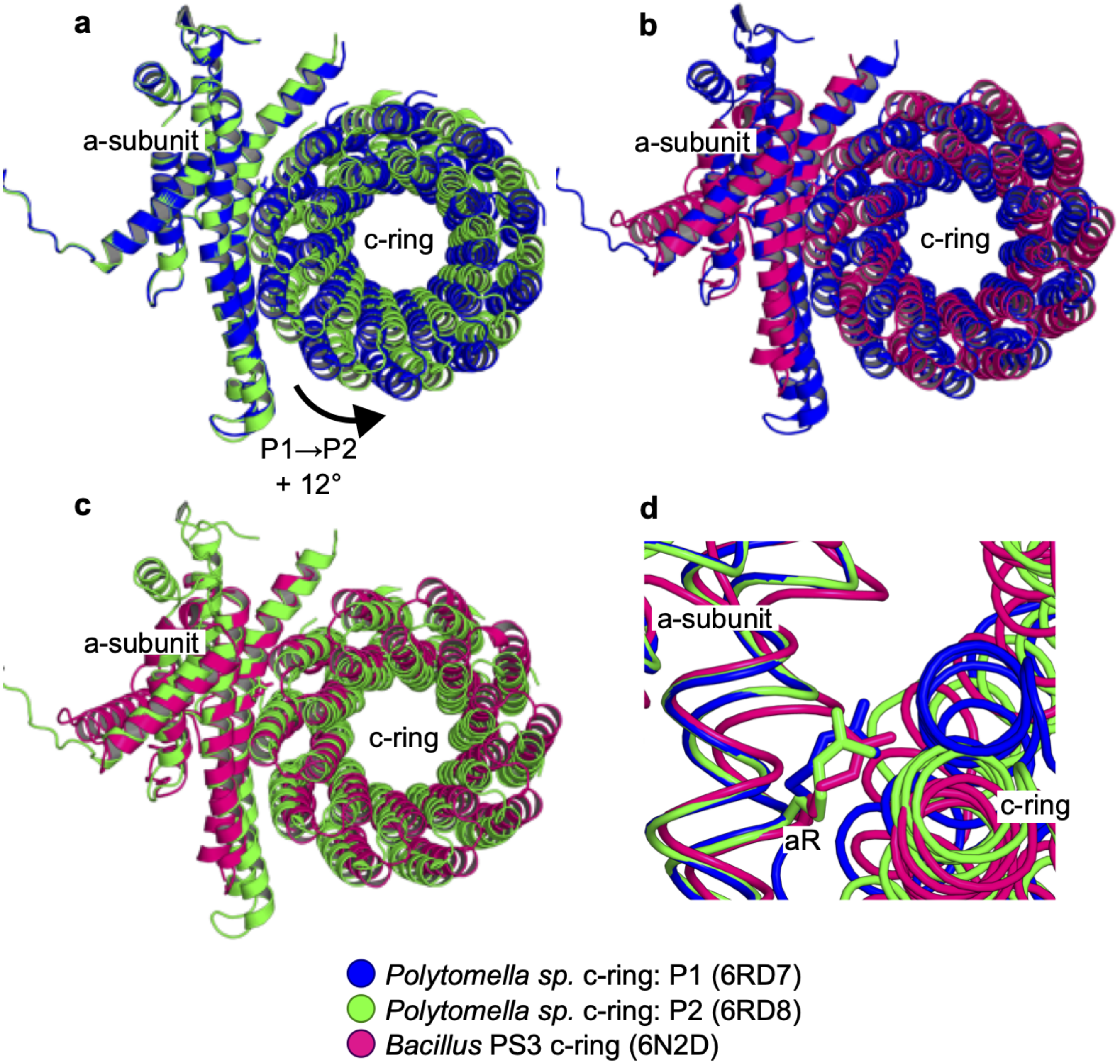

Fig. S10. Comparison of c-ring positions in cryo-EM structures of *Polytomella sp.* and *Bacillus sp. PS3* ATP synthases. To isolate c-ring movement, structures are aligned on the structurally conserved a-subunit using Pymol (32) (a) Alternate c-ring positions P1 (6RD7/6RD9, blue) and P2 (6RD8, green) seen in *Polytomella sp.* ATP synthase (1). P2 is rotated forward by $\approx 12^{\circ}$ with respect to P1 used as a reference. (b) Comparison of c-ring positions between *Polytomella sp.* P1 structure (6RD7/6RD9, blue) and *Bacillus PS3* structure (6N2D, hotpink) (33) shows that the *Bacillus* c-ring is rotated forward with respect to P1. (c) Comparison of c-ring positions between *Polytomella sp.* P2 structure (6RD8, green) and *Bacillus PS3* structure (6N2D, hotpink) shows that the *Bacillus* c-ring occupies approximately the same position as P2. (d) Close-up on the conserved a-subunit Arginine residue aR (aR239 in *Polytomella sp.*, aR169 in *Bacillus*) showing that the aR CA atoms are superimposed. This illustrates that the structural alignment onto the a-subunit is meaningful and can be used to compare c-ring rotational states across species.

Subunit	Chain	Residues
c_{10}	A to J	54 to 127
a	M	95 to 327
ASA1	1	520 to 617
ASA3	3	154 to 321
ASA5	5	1 to 61
ASA6	6	28 to 151
ASA8	8	2 to 89
ASA9	9	1 to 97
ASA10	0	2 to 82

Table S1. F_o region minimal model

Protonation state	Exploratory run	Stratified run	Total simulation time
OOH	$8.2\,\mu s$	$15 \times 1.64 = 24.6\,\mu s$	$32.8\,\mu s$
OHH	$6\,\mu s$	$12 \times 2.48 = 29.7\,\mu s$	$35.7\,\mu s$

Table S2. List and length of eABF simulations.

Protonation state	Simulation time
OOH with protonated aE288	$64 \times 0.24 = 15.36\,\mu s$
OOH with deprotonated aE288 (identical to OOH)	$64 \times 0.24 = 15.36\,\mu s$

Table S3. List and length of c-ring restrained simulations.

Lipid	Proportion
Cardiolipin	$16\,\%$
POPE	$37\,\%$
POPC	$41\,\%$
POPI	$6\,\%$

Table S4. Lipid composition of the membrane

State	Label	Protonation states
Pre-protonation	OOH	cE111J: charged, cE111A: charged, cE111B: protonated
Post-protonation	OHH	cE111J: charged, cE111A: protonated, cE111B: protonated

Table S5. Protonation states of the c-ring

References

1. BJ Murphy, et al., Rotary Substates of Mitochondrial ATP Synthase Reveal the Basis of flexible F1-Fo coupling. *Science* **364**, eaaw9128 (2019).
2. N Eswar, et al., Comparative Protein Structure Modeling Using Modeller. *Curr. Protoc. Bioinforma.* **15**, 5.6.1–5.6.30 (2006).
3. N Klusch, BJ Murphy, DJ Mills, Ö Yildiz, W Kühlbrandt, Structural basis of proton translocation and force generation in mitochondrial ATP synthase. *eLife* **6**, e33274 (2017).
4. D Bashford, M Karplus, Multiple-site titration curves of proteins: An analysis of exact and approximate methods for their calculation. *J. Phys. Chem.* **95**, 9556–9561 (1991).
5. CA Fitch, G Platzer, M Okon, B Garcia-Moreno E., LP McIntosh, Arginine: Its pKa value revisited. *Protein Sci.* **24**, 752–761 (2015).
6. NA Baker, D Sept, S Joseph, MJ Holst, JA McCammon, Electrostatics of nanosystems: Application to microtubules and the ribosome. *Proc. Natl. Acad. Sci. U.S.A.* **98**, 10037–10041 (2001).
7. B Rabenstein, EW Knapp, Calculated pH-Dependent Population and Protonation of Carbon-Monoxy-Myoglobin Conformers. *Biophys. J.* **80**, 1141–1150 (2001).
8. G Kieseritzky, EW Knapp, Optimizing pKA computation in proteins with pH adapted conformations. *Proteins: Struct. Funct. Bioinforma.* **71**, 1335–1348 (2007).
9. SE Horvath, G Daum, Lipids of mitochondria. *Prog. Lipid Res.* **52**, 590–614 (2013).
10. S Jo, T Kim, W Im, Automated Builder and Database of Protein/Membrane Complexes for Molecular Dynamics Simulations. *PLOS ONE* **2**, e880 (2007).
11. S Jo, T Kim, VG Iyer, W Im, CHARMM-GUI: A web-based graphical user interface for CHARMM. *J. Comput. Chem.* **29**, 1859–1865 (2008).
12. EL Wu, et al., CHARMM-GUI Membrane Builder toward realistic biological membrane simulations. *J. Comput. Chem.* **35**, 1997–2004 (2014).
13. J Lee, et al., CHARMM-GUI Input Generator for NAMD, GROMACS, AMBER, OpenMM, and CHARMM/OpenMM Simulations Using the CHARMM36 Additive Force Field. *J. Chem. Theory Comput.* **12**, 405–413 (2016).
14. J Huang, et al., CHARMM36m: An improved force field for folded and intrinsically disordered proteins. *Nat. Methods* **14**, 71–73 (2017).
15. RH Stote, M Karplus, Zinc binding in proteins and solution: A simple but accurate nonbonded representation. *Proteins: Struct. Funct. Genet.* **23**, 12–31 (1995).
16. MJ Abraham, et al., GROMACS: High performance molecular simulations through multi-level parallelism from laptops to supercomputers. *SoftwareX* **1–2**, 19–25 (2015).
17. G Bussi, D Donadio, M Parrinello, Canonical sampling through velocity rescaling. *J. Chem. Phys.* **126**, 014101 (2007).
18. J Hénin, Fast and Accurate Multidimensional Free Energy Integration. *J. Chem. Theory Comput.* **17**, 6789–6798 (2021).
19. A Lesage, T Lelièvre, G Stoltz, J Hénin, Smoothed Biasing Forces Yield Unbiased Free Energies with the Extended-System Adaptive Biasing Force Method. *J. Phys. Chem. B* **121**, 3676–3685 (2017).
20. G Fiorin, ML Klein, J Hénin, Using Collective Variables to Drive Molecular Dynamics Simulations. *Mol. Phys.* **111**, 3345–3362 (2013).
21. F Blanc, et al., An Intermediate Along the Recovery Stroke of Myosin VI Revealed by X-ray Crystallography and Molecular Dynamics. *Proc. Natl. Acad. Sci. U.S.A.* **115**, 6213–6218 (2018).
22. J Wereszczynski, JA McCammon, Nucleotide-dependent mechanism of Get3 as elucidated from free energy calculations. *Proc. Natl. Acad. Sci. U.S.A.* **109**, 7759–7764 (2012).
23. D Branduardi, G Bussi, M Parrinello, Metadynamics with Adaptive Gaussians. *J. Chem. Theory Comput.* **8**, 2247–2254 (2012).
24. E Jones, T Oliphant, P Peterson, Scipy: Open source scientific tools for python. (2001).
25. P Virtanen, et al., SciPy 1.0: Fundamental algorithms for scientific computing in Python. *Nat. Methods* **17**, 261–272 (2020).
26. F Pedregosa, et al., Scikit-learn: Machine Learning in Python. *J. Mach. Learn. Res.* **12**, 2825–2830 (2011).
27. N Michaud-Agrawal, EJ Denning, TB Woolf, O Beckstein, MDAnalysis: A toolkit for the analysis of molecular dynamics simulations. *J. Comput. Chem.* **32**, 2319–2327 (2011).
28. R Gowers, et al., MDAnalysis: A Python Package for the Rapid Analysis of Molecular Dynamics Simulations in *Python in Science Conference.* (Austin, Texas), pp. 98–105 (2016).
29. AA Hagberg, DA Schult, PJ Swart, Exploring Network Structure, Dynamics, and Function using NetworkX in *Python in Science Conference.* (2008).
30. BJ Siwick, MJ Cox, HJ Bakker, Long-Range Proton Transfer in Aqueous Acid-Base Reactions. *J. Phys. Chem. B* **112**, 378–389 (2008).
31. MJ Cox, RLA Timmer, HJ Bakker, S Park, N Agmon, Distance-Dependent Proton Transfer along Water Wires Connecting Acid-Base Pairs. *J. Phys. Chem. A* **113**, 6599–6606 (2009).
32. Schrödinger, LLC., *The PyMOL Molecular Graphics System.*
33. H Guo, T Suzuki, JL Rubinstein, Structure of a bacterial ATP synthase. *eLife* **8**, e43128 (2019).
